# Estimation of required sample size for external validation of risk models for binary outcomes

**DOI:** 10.1177/09622802211007522

**Published:** 2021-04-21

**Authors:** Menelaos Pavlou, Chen Qu, Rumana Z Omar, Shaun R Seaman, Ewout W Steyerberg, Ian R White, Gareth Ambler

**Affiliations:** 1 Department of Statistical Science, University College London, UK; 2 MRC Biostatistics Unit, Institute of Public Health, University of Cambridge, Cambridge, UK; 3 Department of Biomedical Data Sciences, Leiden University Medical Center, Leiden, Netherlands; 4 MRC Clinical Trials Unit, University College London, London, UK

**Keywords:** Sample size calculation, prediction model, C-statistic, discrimination, calibration

## Abstract

Risk-prediction models for health outcomes are used in practice as part of clinical decision-making, and it is essential that their performance be externally validated. An important aspect in the design of a validation study is choosing an adequate sample size. In this paper, we investigate the sample size requirements for validation studies with binary outcomes to estimate measures of predictive performance (C-statistic for discrimination and calibration slope and calibration in the large). We aim for sufficient precision in the estimated measures. In addition, we investigate the sample size to achieve sufficient power to detect a difference from a target value. Under normality assumptions on the distribution of the linear predictor, we obtain simple estimators for sample size calculations based on the measures above. Simulation studies show that the estimators perform well for common values of the C-statistic and outcome prevalence when the linear predictor is marginally Normal. Their performance deteriorates only slightly when the normality assumptions are violated. We also propose estimators which do not require normality assumptions but require specification of the marginal distribution of the linear predictor and require the use of numerical integration. These estimators were also seen to perform very well under marginal normality. Our sample size equations require a specified standard error (SE) and the anticipated C-statistic and outcome prevalence. The sample size requirement varies according to the prognostic strength of the model, outcome prevalence, choice of the performance measure and study objective. For example, to achieve an SE < 0.025 for the C-statistic, 60–170 events are required if the true C-statistic and outcome prevalence are between 0.64–0.85 and 0.05–0.3, respectively. For the calibration slope and calibration in the large, achieving SE < 0.15   would require 40–280 and 50–100 events, respectively. Our estimators may also be used for survival outcomes when the proportion of censored observations is high.

## 1 Introduction

Clinical risk-prediction models are used to predict the risk of either having a health outcome (diagnostic models) or developing a health outcome in the future (prognostic models) using information on patient characteristics. These models are often developed using a regression model that associates the outcome to patient characteristics, the predictor variables. For binary outcomes, a logistic regression model is commonly used. The model is fitted to the development data to estimate the regression coefficients which can then be used to predict the outcome in new patients. Risk-prediction models (hereafter ‘risk models’) have important clinical applications; for example, they are used for clinical decision-making and the clinical management of patients,^1–3^ to assess the performance of hospitals and clinicians by policy makers^4^ and in precision medicine to identify patient subgroups for targeted treatment.^5^

Given the important role of risk models in health care, it is essential to validate risk models, i.e. to assess their predictive performance in either the data used for model development (internal validation) or in a new dataset (external validation). Typically, in external validation, the risk model is used to obtain predictions for patients in a new dataset, and the quality of these predictions is assessed using measures of predictive performance, for example, measures of calibration, such as the calibration slope and calibration in the large, and measures of prognostic strength (also called discrimination), such as the C-statistic. A crucial aspect of designing an external validation study is deciding how large the sample size should be. A systematic review of published external model validation studies found that just under half of the studies evaluated models using datasets with fewer than 100 events.^6^

Some broad recommendations have been made regarding the sample size for external validation studies for binary and survival outcomes. Harrell et al.^7^ suggested that at least 100 events should be available in the validation data. Vergouwe et al.^8^ suggested at least 100 events and 100 non-events are required in the validation dataset for binary outcomes. Their recommendation was based on the sample size required to detect a statistically significant difference between the estimate of the performance measure and a pre-specified value with 80% power and 5% significance level (for example, assuming a difference of 0.1 for the C-statistic). They used the estimated variance of the performance measure from the development data to calculate the sample size assuming that the outcome prevalence in the development and validation datasets is the same. When this assumption was unlikely to hold, they suggested using simulation to estimate the variance corresponding to a different prevalence in the validation data. Peek et al.^9^ concluded that ‘substantial sample sizes’ are required for external validation studies to reliably test for lack of model fit (assessed using the calibration slope and Hosmer–Lemeshow test statistic) based on the resampling of large datasets and examining the variability of performance measures. They suggested avoiding the use of test-based approaches when assessing the predictive performance of models because of the large sample size requirements for the validation data. Collins et al.^10^ used resampling methods to calculate the variance of performance measures and recommend a minimum of 100 events, and preferably 200 events or more, to obtain unbiased and precise estimates. Snell et al.^11^ also used simulation to explore the sample size requirements for precision-based sample size calculations and considered a wide range of scenarios.

We focus on the two most common scenarios where the objective is either to calculate the required sample size to obtain an estimate of a measure of predictive performance with a desired level of precision, or to provide sufficient power to detect a difference in the estimate of a measure of predictive performance from a target value. The main aim of this paper is to derive formulae that can be used to calculate the sample size for external validation studies, by only making a few assumptions regarding the features of the validation dataset and using information about the anticipated population values of the C-statistic and outcome prevalence, quantities that can be obtained from previous studies. Moreover, since the sample size requirements may be affected by the prognostic strength of the model, a factor that has been linked to the sample size requirements for model development,^12,13^ we also investigate how the prognostic strength of the model and the prevalence of the outcome in the validation data influence the sample size requirements.

The structure of the paper is as follows. We start with the case where the outcome in a prediction model is binary. In Section 2, we introduce the measures of predictive performance considered in this paper and describe the possible objectives of an external validation study. In Section 3, we derive formulae for the variance of the estimated values of the C-statistic, calibration slope and calibration in the large that do not require any patient-level information. In Section 4, we use our variance formulae to derive formulae for precision- and power-based calculations and discuss how model strength and outcome prevalence affect sample size requirements. We use a simulation study in Section 5 to evaluate our variance and sample size formulae when the assumptions are met and under reasonable departures from these assumptions. Section 6 discusses alternative approaches to sample size calculation that may be used when the outcome in the prediction model is a survival time which may be subject to censoring. In Section 7, we demonstrate the application of the methods in a scenario with real data, and Section 8 provides a discussion.

## 2 Measures of predictive performance and criteria for sample size calculation

The predictive performance of a risk model in an external validation study is typically assessed using measures of calibration and discrimination. The calculation of these performance measures is based on the observed outcomes and the predicted probabilities in the validation data. These predicted probabilities are usually calculated using regression coefficients estimated in the development data and the predictor information in the validation data but could also be obtained from more recent modelling approaches such as random forests, support vector machines, neural networks and other machine learning techniques.^14^

The most popular measure of model discrimination when the outcome is binary is the C-statistic, which measures the ability to separate individuals who experience the event of interest from those who do not. Considering two discordant patients, i.e. one who experiences the event and one who has not, the C‐statistic is the probability that the patient who experiences the event has a higher predicted risk than the patient who does not. A value of 0.5 suggests that the model has no discriminatory ability, while a value of 1 suggests that the model can discriminate perfectly between patients who experience events and those who do not. A risk model with a higher value of the C-statistic has a higher *model strength* than a model with a lower value of the C-statistic.

Calibration is often assessed using the calibration in the large and calibration slope. For the calibration slope, the binary outcome is regressed on the linear predictor in a logistic regression model, and the coefficient of the linear predictor in this regression is the calibration slope.^15^ A slope of one suggests perfect calibration, a slope of less than one suggests overfitting and a slope greater than one suggests underfitting. For the calibration in the large, a similar regression model is considered as for the calibration slope, but with the coefficient of the linear predictor fixed to the value of one. The intercept term in this model is the calibration in the large. A calibration in the large of zero suggests that the proportion of events is equal to the mean of the predicted probabilities. A negative (positive) value suggests that the predicted probabilities are on average higher (lower) than the proportion of events. In this paper, we investigate the sample size requirements for validation studies when the main measures of predictive performance are the C-statistic, calibration slope and calibration in the large.

We consider two criteria to calculate the sample size for an external validation study based on different clinical aims:

- Precision-based: the aim is to obtain an estimate of a measure of predictive performance, for example, the C-statistic, with a certain degree of precision expressed by the size of the standard error (SE) (or equivalently, the width of the confidence interval).
- Power-based: the aim is to detect whether the value of a measure of predictive performance (for example, the C-statistic) is significantly different from a pre-specified target value (e.g. C=0.70 ) with sufficient power (e.g. 80%) and a fixed Type I error (e.g. 5%).

Previous studies investigating sample size requirements for validation studies used simulation or resampling-based methods to make some broad sample size recommendations based on estimating the variance of the estimated performance measures. In contrast, we aim to obtain formulae for the variance of the estimated performance measures as a function of the sample size and the true population values of the C-statistic and outcome prevalence. In practice, values for the latter two quantities are not known and anticipated population values, i.e. anticipated values in the population in which the validation study will be carried out, may be obtained from previous studies or expert clinical opinion. These formulae allow us to perform precision- or power-based sample size calculations for a particular study without the need to simulate data, thus entailing less computation.

In the next section, we obtain formulae for the variance of the estimated performance measures that do not require any patient-level data. Based on these, we then obtain formulae to perform precision- and power-based sample size calculations.

## 3 Formulae for the variance of the estimated measures of predictive performance and for sample size calculations for binary outcomes

### 3.1 Variance of the estimated C-statistic

Let Y denote the binary outcome and η the linear predictor (predicted log-odds) when logistic regression is used. We let F denote the distribution of the linear predictor in the population and $\pi \left(\eta \right)=P\left(Y=1\;\right|\;\eta ){=\left(1+e^{-\eta }\right)}^{-1}$ . Let $\left(Y_{1},\eta_{1}),\ldots ,(Y_{n},\eta_{n}\right)$ denote a random sample of size n from the population we wish to validate a risk model on, where n denotes the size of the validation dataset. Let $n_{0}$ and $n_{1}\;$ denote the number of subjects with Y = 0 and *Y = 1*, respectively. Subjects with *Y = 1* have experienced the event of interest and are called ‘cases’, while subjects with *Y = 0* have not experienced the event and are called ‘controls’. Given a pair of subjects $\left(i,\;j\right)\;$ let $\eta_{i}^{\left(1\right)},\;i=1,\ldots ,\;n_{1}\;$ and $\eta_{j}^{\left(0\right)}$ , $\;j=1,\ldots ,\;n_{0}$ denote the linear predictor of the *i*th case and the *j*th control.

The C-statistic can be defined as

(1) $$C=\mathrm{Pr}\left(\eta_{I}^{\left(1\right)}>\eta_{J}^{\left(0\right)}\;\right)+\frac{1}{2}\mathrm{Pr}\left(\eta_{I}^{\left(1\right)}=\eta_{J}^{\left(0\right)}\right)\;$$

where I is the index of a randomly chosen case and J is the index of a randomly chosen control.

An estimator for the C-statistic is

(2) $$\hat{C}=\frac{1}{n_{0}n_{1}}\sum_{i}\sum_{j}I({\hat{\eta }}_{i}^{\left(1\right)},{\hat{\eta }}_{j}^{\left(0\right)})\;\;$$

where the summation $\sum_{i}\;$ is over the cases and $\sum_{j}\;$ is over the controls. The indicator variable $I({\hat{\eta }}_{i}^{\left(1\right)},{\hat{\eta }}_{j}^{\left(0\right)})$ is defined as follows

$$I\left({\hat{\eta }}_{i}^{\left(1\right)},{\hat{\eta }}_{j}^{\left(0\right)}\right)=\left\{\;\begin{array}{ll} 1 & if\;{\hat{\eta }}_{i}^{\left(1\right)}>{\hat{\eta }}_{j}^{\left(0\right)}\;\\ 1/2 & if\;{\hat{\eta }}_{i}^{\left(1\right)}={\hat{\eta }}_{j}^{\left(0\right)}\;\\ 0 & if\;{\hat{\eta }}_{i}^{\left(1\right)}<{\hat{\eta }}_{j}^{\left(0\right)} \end{array}\right.$$

Several methods have been proposed for the estimation of the variance of the C-statistic. Simulation studies^16^ have shown that the variance estimator proposed by DeLong et al.^17^ performs best. DeLong’s variance estimator is given by

(3) $${\hat{\mathrm{var}}}_{\mathrm{DL}}\left(\hat{C}\right)=\;\frac{S_{10}}{n_{1}}\;+\frac{\;S_{01}}{n_{0}}$$

where

$$S_{10}=\frac{1}{n_{1}}\;\sum_{i}{\left(\frac{\sum_{j}I\left({\hat{\eta }}_{i}^{\left(1\right)},{\hat{\eta }}_{j}^{\left(0\right)}\right)}{n_{0}}-\hat{C}\right)}^{2}\;,\;{\;S}_{01}=\frac{1}{n_{0}}\;\sum_{j}{\left(\frac{\sum_{i}I\left({\hat{\eta }}_{j}^{\left(0\right)},{\hat{\eta }}_{i}^{\left(1\right)}\right)}{n_{1}}-\hat{C}\;\right)}^{2}\;$$

and $\sum_{i}\;$ sums over the cases and $\sum_{j}\;$ sums over the controls. DeLong’s formula requires knowledge of the values of the linear predictor and binary outcome for every patient in the study. Hence, if DeLong’s formula is to be used in sample size calculations for a validation dataset for which data have not been collected yet, simulation is required.

An asymptotic approximation to the variance of $\hat{C}$ can be obtained from DeLong’s variance estimator (see Supplementary Material 1) as

(4) $${\hat{\mathrm{var}}}_{NI}\left(\hat{C}\right)=\frac{1}{n}\times \frac{\;\left(1-p\right)E_{\eta^{\left(1\right)}}\left(K^{2}\left(\eta^{\left(1\right)}\right)\right)+pE_{\eta^{\left(0\right)}}{\left(1-G\left(\eta^{\left(0\right)}\right)\right)}^{2}}{p(1-p)}$$

where K and G are the cumulative distribution functions of the linear predictor for the controls and cases, respectively. So, $K\left(\eta^{\left(1\right)}\right)=P\left(\eta^{\left(0\right)}<\eta^{\left(1\right)}\right)$ and $G\left(\eta^{\left(0\right)}\right)=P\left(\eta^{\left(1\right)}<\eta^{\left(0\right)}\right)$ .

If it is assumed that the marginal distribution, F,  of the linear predictor is known and the model for π(η) is well calibrated, the distribution of the linear predictor for the cases and controls, respectively, has been given by Gail and Pfeiffer^18^ as

(5) $$G\left(x\right)=P\left(\eta \leq x\;\right|\;Y=1)=P\left(\eta^{\left(1\right)}\leq x\right)=\frac{\int_{-\infty }^{x}\pi \left(\eta \right)dF\left(\eta \right)}{\int_{-\infty }^{\infty }\pi \left(\eta \right)dF\left(\eta \right)}\;\mathrm{and}\;$$

(6) $$K\left(x\right)=P\left(\eta \leq x\;\right|\;Y=0)=P\left(\eta^{\left(0\right)}\leq x\right)=\frac{\int_{-\infty }^{x}\left(1-\pi \left(\eta \right)\right)dF\left(\eta \right)}{\int_{-\infty }^{\infty }\left(1-\pi \left(\eta \right)\right)dF\left(\eta \right)}\;.\;$$

The cumulative distribution functions of $\eta^{\left(1\right)}$ and $\eta^{\left(0\right)}$ can be computed using numerical integration and then can be used to compute $E_{\eta^{\left(1\right)}}\left(K^{2}\left(\eta^{\left(1\right)}\right)\right)$ and $E_{\eta^{\left(0\right)}}{\left(1-G\left(\eta^{\left(0\right)}\right)\right)}^{2}$ , also by numerical integration. So, provided that the marginal distribution of the linear predictor is available, with the aid of numerical integration, and using relationships (5) and (6), one can compute analytically the variance in (4) without using any individual-level data.

#### Assumption 1 – Marginal normality: η∼N (μ, σ^2^)

In practice, risk models most often include a number of continuous and categorical predictors, and, unless this number is very small or there are only binary predictors with extreme prevalences, the distribution of η  is likely to be approximately marginally Normal.

In applying equation (4) under the assumption of marginal normality, values for the parameters of μ and $\sigma^{2}$ need to be chosen to match the anticipated values of the outcome prevalence and C-statistic. To avoid the use of simulation in choosing suitable values for μ and $\sigma^{2}$ , we obtain in Supplementary Material 1 the following expressions for μ and $\sigma^{2}$

(7) $$\mu \approx \left(2p-1\right){\left(\Phi^{-1}(C)\right)}^{2}+\log\left(\frac{p}{1-p}\right)\;$$

(8) $$\sigma^{2}\approx 2\;{\left(\Phi^{-1}(C)\right)}^{2}\left(p^{2}+{\left(1-p\right)}^{2}\right)\;$$

that correspond approximately to the required anticipated values of C and p.  We also show that the approximation works very well for a wide range of values of C and p (within 1.5% of the required anticipated values in all scenarios).

In Section 5, we use simulation to study the performance of (4) under the assumption of marginal normality, and in Supplementary Material 3, we provide code to compute ${\mathrm{var}}_{NI}\left(\hat{C}\right)$ .

#### Closed-form formula for the variance of the estimated C-statistic

If, instead, the distribution of the linear predictor in cases and controls is assumed to be known, one can obtain the expectations needed in (4) and hence estimate the variance $\mathrm{of}\;\hat{C}$ . Assuming that the conditional distribution of the linear predictor given the outcome is Normal, we obtain a simple estimator of the variance of $\hat{C}\;$ that does not depend on patient-level data.

#### Assumption 2 – Conditional normality: η|Y∼N (μ_Y_, σ_Y_^2^), Y=0, 1.

Under Assumption 2, the C-statistic can be approximated^19^ by $\;C=\Phi \left(\frac{{\mu_{1}-\mu }_{0}}{\sigma_{0}^{2}+\sigma_{1}^{2}\;}\right),\;$ and K and G are the cumulative distribution functions of the Normal distribution with parameters $\mu_{Y}\;\mathrm{and}\;\sigma_{Y}^{2},\;\mathrm{for}\;Y=0,\;1$ . In Supplementary Material 1, we approximate (4) to obtain a simple formula for the variance of $\hat{C}$ that only depends on the sample size , the true value of the C-statistic and the outcome prevalence.

The formula is

(9) $${\hat{\mathrm{var}}}_{\mathrm{app}}\left(\hat{C}\right)=\frac{1}{n}\times \frac{C-2\;T\left({\;\Phi }^{-1}\left(C\right),\frac{1}{\sqrt{3}}\right)-C^{2}}{p(1-p)}\;$$

where p=P(Y=1) denotes the outcome prevalence, Φ the cumulative distribution function of the standard Normal distribution and  T is Owen’s^20^ T -function which can be calculated, for example, using the function ‘T.owen’ in the R package ‘sn’. In Section 5, we use simulation to assess its performance when Assumption 1 holds and under reasonable departures from this assumption. Importantly, as shown later in Section 5, estimator (9) works very well under Assumption 2 but also under the assumption of marginal Normality for η .

**Figure 1.**
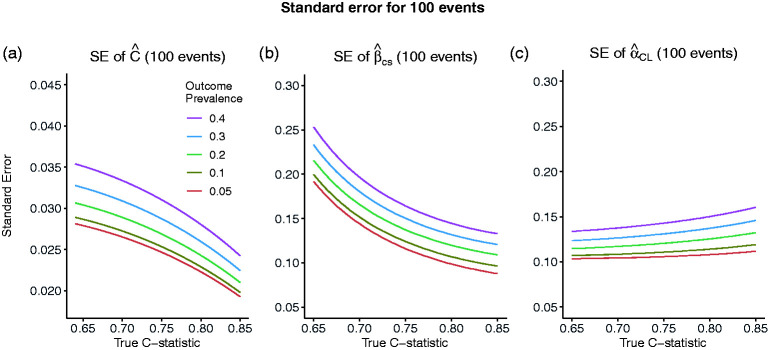
Standard error of the estimated C-statistic (a), calibration slope (b) and calibration in the large (c) as the true value of the C-statistic varies and the number of events is fixed to 100, corresponding to sample sizes of 2000, 1000, 500, 334 and 250 for outcome prevalences of 0.05, 0.1, 0.2, 0.3 and 0.4, respectively. SE: standard error.

Figure 1a shows the relationship between the SE, $\sqrt{{\hat{\mathrm{var}}}_{\mathrm{app}}\left(\hat{C}\right)}$ , of the estimated C-statistic and the true value of the C-statistic for different values of the outcome prevalence. We considered values between 0.64 and 0.85 for the true C to reflect the values typically seen in practice and values of 0.05, 0.1, 0.2, 0.3 and 0.4 for the true outcome prevalence corresponding to sample sizes of 2000, 1000, 500, 334 and 250, respectively, when the number of events is fixed at 100. According to this formula, the SE of $\hat{C}\;$ for a given prevalence and number of events decreases with higher values of the true C and lower values of the true outcome prevalence.

### 3.2 Variance of the estimated calibration slope and calibration in the large

The calibration slope is estimated by fitting the following logistic regression model to the validation data

(10) $$\mathrm{logit}\left(\pi_{i}\right)=\mathrm{logit}\left(P\left(Y_{i}=1|\eta_{i}\right)\right)=\alpha +\beta_{cs}\;\eta_{i},\;i=1,\ldots ,n$$

where $\beta_{cs}$ is the calibration slope and $n=n_{0}+n_{1}$ is the sample size. For a well-calibrated model, ${\hat{\beta }}_{cs}=1.$ Similarly, the calibration in the large is defined as $\alpha_{CL}$ in the model

(11) $$\mathrm{logit}\left(\pi_{i}\right)=\mathrm{logit}\left(P\left(Y_{i}=1|\eta_{i}\right)\right)=\alpha_{CL}+\;\eta_{i},\;i=1,\ldots ,n.$$

This is equivalent to model (10) with $\beta_{cs}\;$ fixed at 1. For a well-calibrated model with respect to the calibration in the large, ${\hat{\alpha }}_{CL}=0.$ The calibration in the large in model (11) can be obtained by fitting a logistic regression model for the binary outcome which includes the linear predictor as an offset term.

Assuming models (10) and (11), an asymptotic approximation for the variances of ${\hat{\alpha }}_{CL}$ and ${\hat{\beta }}_{cs}$ can be obtained from the inverse of Fisher’s information as

(12) $${\hat{\mathrm{var}}}_{NI}\left({\hat{\alpha }}_{CL}\right)=\frac{1}{n}\times \frac{1}{E\left(W\right)}\;$$

(13) $${\;\hat{\mathrm{var}}}_{NI}({\hat{\beta }}_{CS})=\frac{1}{n}\times \frac{E(W)}{E(W)E(W\eta^{2})-E^{2}(W\eta )}$$

where ${W=\pi \left(1-\pi \right)\;\mathrm{and}\;\pi =\left(1+e^{-\eta }\right)}^{-1}.\;$ For a given sample of size n, estimators of (12) and (13) are

$$\hat{\mathrm{var}}\left({\hat{\alpha }}_{CL}\right)=\frac{1}{\sum_{i}w_{i}}\;\mathrm{and}\;\hat{\mathrm{var}}({\hat{\beta }}_{CS})=\frac{\sum_{i}w_{i}}{(\sum_{i}w_{i})\left(\sum_{i}w_{i}\eta_{i}^{2}\right)-{\left(\sum_{i}w_{i}\eta_{i}\right)}^{2}}$$

Assuming that η has a known distribution, the variances in (12) and (13) can be obtained by computing the expectations $E\left(W\right),\;E(W\eta^{2})$ and $E^{2}(W\eta )$ using numerical integration.

For example, if $\eta ∼N\left(\mu ,\;\sigma^{2}\right),$

$$E\left(W\eta \right)=\int_{-\infty }^{\infty }{\left(1+e^{-\eta }\right)}^{-1}\left(1-{\left(1+e^{-\eta }\right)}^{-1}\right)f\left(\eta \right)d\eta$$

where f(η) is the Normal density function. $E(W\eta^{2})$ and E(W) can be computed in a similar manner. The performance of (12) and (13) under the assumption of marginal normality for η is studied in Section 5. The R code to compute the relevant expectations using the function ‘integrate’ is provided in Supplementary Material 3.

Figure 1b and c, similar to Figure 1a, shows the relationship between the SEs $\sqrt{{\hat{\mathrm{var}}}_{\mathrm{NI}}({\hat{\beta }}_{CS})}$ and $\sqrt{{\hat{\mathrm{var}}}_{\mathrm{NI}}\left({\hat{a}}_{CL}\right)}$ and the true value of C when the number of events is fixed. The distribution of the linear predictor is assumed to be marginally Normal. The SE of the estimated calibration slope decreases with higher C and lower prevalence. In comparison to Figure 1a, it can also be seen that for a given prevalence, the SE of the estimated calibration slope declines faster than the SE of the estimated C-statistic as the true C increases. The SE of the estimated calibration in the large decreases with lower prevalence and, contrary to the calibration slope, it increases with increasing C-statistic, although the increase is very gradual.

#### Closed-form variance estimators for calibration slope and calibration in the large

To obtain a simple formulae for the variance of the estimated calibration in the large that is free from patient-level information and avoids use of numerical integration, we make the assumption that the marginal distribution of the linear predictor is Normal (Assumption 1). In Supplementary Material 1, we approximate E(W) in (12) to obtain the following estimator for the variance of the estimated calibration in the large

(14) $${\hat{\mathrm{var}}}_{\mathrm{app}}\left({\hat{\alpha }}_{CL}\right)=\frac{1}{n}\times \tilde{\pi }(1-\tilde{\pi })\left(1+\frac{1}{2}\left(1\;-\;6\tilde{\pi }+6{\tilde{\pi }}^{2}\right)\;\sigma^{2}\right)$$

where

(15) $$\tilde{\pi }={\left(1+e^{-\mu }\right)}^{-1}$$

and μ and $\sigma^{2}$ of the assumed Normal distribution can be obtained by (7) and (8), respectively.

To obtain an analogous formula for the variance of the estimated calibration slope that does not require the use of numerical integration, we assume that the conditional distribution of the linear predictor given Y is Normal and make the additional assumption that the corresponding variances are equal.

#### Assumption 3 – Conditional normality with equal variances: η|Y∼N (μ_Y_, σ^2^), Y=0, 1

Using results from the relationship between the parameters in a logistic regression model and the corresponding linear discriminant analysis (LDA) model,^21–23^ we obtain in Supplementary Material 1 the following formula for the variance of the estimated calibration slope that depends on the sample size, the true value of the C-statistic, the outcome prevalence and the calibration slope

(16) $${\hat{\mathrm{var}}}_{\mathrm{app}}({\hat{\beta }}_{CS})=\frac{\beta_{cs}^{2}}{2\;p\;\left(1-p\right)\;n{\;\Phi }^{-1}{\left(C\right)}^{2}}+\frac{2\;\beta_{cs}^{2}}{n}\;\;$$

where $\beta_{CS}$ denotes the true value of the calibration slope.

In Section 5, we use simulation to evaluate the performance of (14) and (16) when their corresponding assumptions are met and under reasonable departures from these assumptions and also to establish a range of values of C for which the formulae can be reliably used.

## 4 Formulae for precision- and power-based sample size calculations

In this section, we use our variance estimators of Section 3 to obtain formulae for precision and power-based sample size calculations.

### 4.1 Precision-based sample size calculations

The most appropriate approach for sample size calculation for most validation studies is likely to be aimed at obtaining an estimate of a performance measure with reasonable precision, as measured by the size of the SE or the width of the confidence interval. Rearranging equation (9) to perform a precision-based sample size calculation based on the true values of C and p,  and letting the required variance of $\hat{C}$ be $\mathrm{va}r_{\mathrm{req}}\left(\hat{C}\right),\;$ we obtain:

(17) $${\hat{n}}_{\mathrm{req},\mathrm{app}}(C)=\frac{C-2T\left(\Phi^{-1}\left(C\right),\frac{1}{\sqrt{3}}\right)-C^{2}}{p(1-p)\;{\mathrm{var}}_{\mathrm{req}}(\hat{C})\;}\;$$

Similarly, rearranging equations (16) and (14) and letting the required variance of the calibration slope $\beta_{CS}$ and calibration in the large be $\mathrm{va}r_{\mathrm{req}}({\hat{\beta }}_{CS}$ ) and ${\mathrm{var}}_{\mathrm{req}}\left({\hat{\alpha }}_{CL}\right),$ respectively, the required sample sizes are given by

(18) $${\hat{n}}_{\mathrm{req},\mathrm{app}}(\beta_{cs})=\frac{\beta_{CS}^{2}\;}{\mathrm{va}r_{\mathrm{req}}({\hat{\beta }}_{CS})}\left(\frac{1}{4p\left(1-p\right){\;\Phi }^{-1}{\left(C\right)}^{2}\;}+2\right)$$

(19) $${\hat{n}}_{\mathrm{req},\mathrm{app}}\left(\alpha_{CL}\right)=\;\frac{\tilde{\pi }(1-\tilde{\pi })}{\mathrm{va}r_{\mathrm{req}}\left({\hat{\alpha }}_{CL}\right)}\left(1\;+\frac{1}{2}\left(1\;-\;6\tilde{\pi }+6{\tilde{\pi }}^{2}\right)\sigma^{2}\right)$$

where $\tilde{\pi },\;\mu \;\mathrm{and}\;\sigma^{2}$ are obtained from equations (15), (7) and (8). Analogous estimators, ${\hat{n}}_{\mathrm{req},\mathrm{NI}}(C)$ , ${\hat{n}}_{\mathrm{req},\mathrm{NI}}(\alpha_{CL})$ and ${\hat{n}}_{\mathrm{req},\mathrm{NI}}(\beta_{cs})$ are obtained by rearranging equations (4), (12) and (13), respectively.

The closed-form formulae for the sample size require the true values of C, p and $\beta_{CS}$ to be specified. The formulae that require the use of numerical integration also require to specify the marginal distribution of the marginal predictor to correspond to the anticipated values of C and p . The C-statistic and outcome prevalence are study-dependent characteristics, and anticipated population values for these measures may be obtained from previously published studies or expert clinical opinion. For example, the anticipated population value of C may be obtained from the original model development paper or other published risk models in similar topic areas. The true value of $\beta_{CS}$ would typically be assumed to be 1, unless there is an indication that the model is over- or underfitted. The precision-based approach also requires the variance of the estimated performance measure or, equivalently, the width of the confidence interval to be specified. This choice will need to be made by the investigator and will depend on the level of precision considered adequate for a given study.

#### Effect of model strength and outcome prevalence on the sample size/number of events

**Figure 2.**
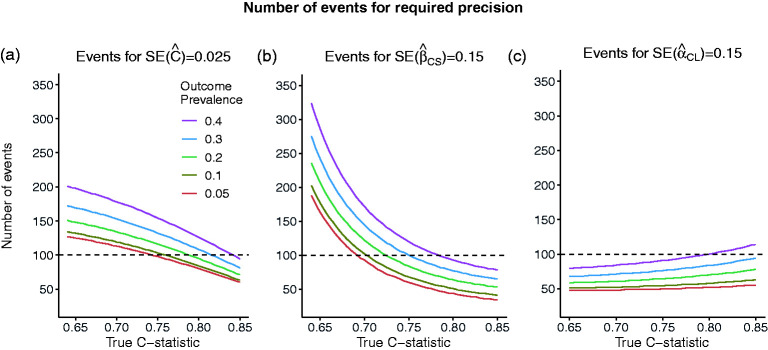
Number of events required to achieve required standard errors of: (a) SE = 0.025 for the estimated C-statistic of 0.025 (width of 95% CI = 0.1) or (b) SE = 0.15 for the estimated calibration slope (width of 95% CI = 0.6) or (c) SE = 0.15 for the estimated calibration in the large, as the true value of the C-statistic and the outcome prevalence varies. SE: standard error.

The effect of model strength and outcome prevalence on the number of events required to achieve the required precision of an estimated measure of predictive performance is illustrated in Figure 2. The number of events, ${\hat{n}}_{\mathrm{req},\mathrm{app}}\left(\hat{C}\right),\;{\hat{n}}_{\mathrm{req},\mathrm{NI}}({\hat{\beta }}_{CS}),\;{\hat{n}}_{\mathrm{req},\mathrm{NI}}\left({\hat{\alpha }}_{CL}\right)$ , required to estimate C with an SE of 0.025 and $\beta_{CS}$ and $\alpha_{CL}\;$ with an SE of 0.15 is shown for a range of outcome prevalences (p=0.05, 0.1, 0.2, 0.3, 0.4) and model strengths (C=0.64 –0.85). The distribution of the linear predictor for ${\hat{n}}_{\mathrm{req},\mathrm{NI}}({\hat{\beta }}_{CS}),\;{\hat{n}}_{\mathrm{req},\mathrm{NI}}\left({\hat{\alpha }}_{CL}\right)$ is assumed to be marginally Normal. For the C-statistic and the calibration slope, the number of events required decreases with higher model strength and lower prevalence. On the other hand, for calibration in the large, the number of events required decreases with lower model strength and increases with higher prevalence. These factors are not taken into consideration in previous sample size recommendations.

For example, we compare the sample size recommendations for the C-statistic, calibration slope and calibration in the large for a given value of outcome prevalence of 10% and different model strengths for  C=0.64, 0.72, 0.8 . For C=0.64 , 1340 patients (rounded up to the nearest 10) and 134 events are required to achieve an $SE(\hat{C})=0.025$ . For C=0.72 and 0.8, the corresponding numbers are 1130 patients (113 events) and 840 patients (84 events), respectively. The sample sizes (number of events) required to estimate the calibration slope with an SE of 0.15, are 2020 (202), 860(86), 510(51) for values of C=0.64, 0.72  and 0.8, respectively. Finally, the sample sizes (number of events) required to estimate the calibration in the large with an SE of 0.15 are 510 (51), 530(53), 580(58) for values of C=0.64, 0.72  and 0.8, respectively. It is noted that when C=0.64 , the required number of events based on C is lower than that based on $\beta_{CS}$ (134 vs. 202), but when C=0.8 , the required number of events based on the calibration slope is smaller (84 vs. 51 events).

### 4.2 Power-based sample size calculations

Power-based sample size calculations are appropriate to investigate whether the performance of an existing risk model holds in a different patient population, for example, in patients from a different country or in patients from a different time-period. For a measure of predictive performance, θ , the null hypothesis is specified as:

$H_{0}:\;\theta =\theta_{0}$ , and the alternative hypothesis can be either

$H_{1}:\;\theta <\theta_{0}$ or $H_{1}:\;\theta >\theta_{0}.$

The power, *1-β* ( β is the Type II error), is the probability of rejecting $H_{0}$ when

- The true value of θ is $\theta_{1}=\theta_{0}+d$ and
- The threshold for rejecting $H_{0}$ has been chosen so that the probability of rejecting $H_{0}$ when the true value of θ is $\theta_{0}$ is α (Type I error or significance level) .

The power-based approach to sample size calculation is particularly relevant for the C-statistic. For example, assuming that the case-mix has remained unchanged, we may wish to show that an existing risk model is outdated, i.e. its predictive performance has deteriorated over time and so $H_{1}:\;C<C_{0}$ . Alternatively, we may wish to demonstrate that a newly developed model has higher discrimination than an established standard, so $H_{1}:\;C>C_{0}.\;$ Based on a one-sided test, the sample size required to detect a statistically significant difference *d* with power *1-β* at the significance level α is

(20) $$\hat{n}(C_{0},d)=\;\frac{{\left(z_{1-\alpha }\times s_{0}+\;z_{\beta }\times s_{1}\right)}^{2}\;}{\left(p-p^{2}\right){\;d}^{2}}$$

where

$$s_{0}=\sqrt{C_{0}-2T\left(\Phi^{-1}\left({\;C}_{0}\right),\frac{1}{\sqrt{3}}\right)-{{\;C}_{0}}^{2}},\;s_{1}=\;\sqrt{C_{1}-2T\left(\Phi^{-1}\left({\;C}_{1}\right),\frac{1}{\sqrt{3}}\right)-{{\;C}_{1}}^{2}}.$$

An analogous result can be derived for the calibration slope should this be required.

**Figure 3.**
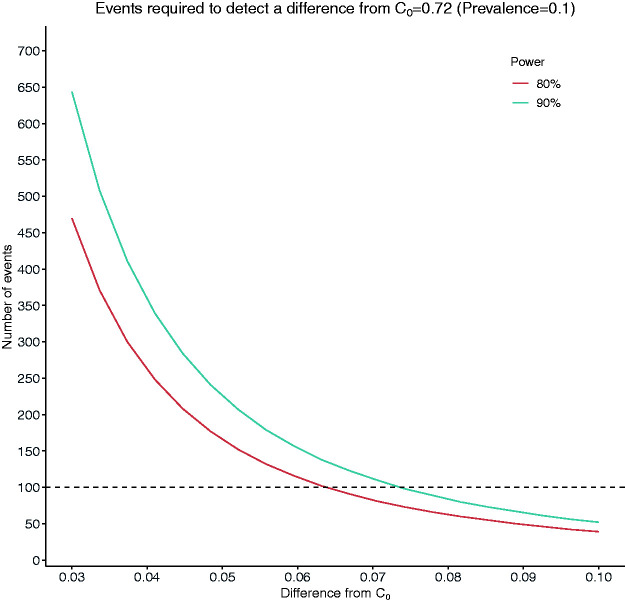
Number of events required to detect a difference of magnitude between 0.03 and 0.1 from a target value of *C* = 0.72 (C_1_ = C_0_ + d).

Figure 3 shows the number of events required to detect a difference, d,  ranging from 0.03 to 0.1 when $C_{0}=0.72$ and $C_{1}=C_{0}+d$ , with 80% or 90% power at the 5% significance level assuming a prevalence of 10%. With a sample size of 1000 patients (100 patients), it is only possible to detect a statistically significant difference of 0.063 for the C-statistic with 80% power. Also, over 4500 patients (450 events) are required to detect a difference of 0.03 with 80% power.

## 5 Simulation study

We use simulation to assess the performance of the variance and sample size formulae of Sections 3 and 4. We plan and report our simulation studies using the structure proposed by Morris et al.^24^ which involves defining aims, data-generating mechanisms (DGMs), estimands/targets, methods and performance measures. All simulations were performed using the R software. The main code for simulations can be found in https://github.com/c-qu/samplesize-validation and in the Supplementary Material 3.

### 5.1 Simulation settings

#### Aims

1. Our variance estimators for the C-statistic, calibration slope and calibration in the large rely on approximations and assumptions. So we assess their performance first in settings where the assumptions hold to assess the quality of the approximations and then where there are departures from the assumptions.
2. We assess the performance of our sample size estimators for (a) precision- and (b) power-based sample size calculations in the same settings.

#### Data-generating mechanisms

We consider four DGMs that correspond to different degrees of departure from the assumptions. The distribution of the linear predictor is assumed to be:

1. Conditionally Normal given the outcome with the corresponding variances being equal.
2. Conditionally Normal given the outcome with the corresponding variances being unequal. This results in a violation of the assumption of equal variances required by the formula for the calibration slope.
3. Marginally Normal. This results in mild violation of both the assumption of conditional normality given the outcome and the assumption of equal variances.
4. Marginally non-Normal in a way that results in marked violation of the assumption of marginal normality and both the assumption of conditional normality given the outcome and the assumption of equal variances.

The technical details of the data-generating process for DGMs 1–4 are presented in the Supplementary Material 2.

#### Parameter values

For all DGMs and aims, we consider a range of values for C and p : C∈{0.64, 0.72, 0.8, 0.85, 0.9} and p∈ {5%, 10%, 30%}. For Aim 1, the number of events $n_{e}\in \{50,\;100,\;150,\;200,\;400\}.\;$ For Aim 2, the required SE for $\hat{C}$ , $SE_{\mathrm{req}}\left(\hat{C}\right)\in$ {0.0125, 0.025} and the required errors for ${\hat{\beta }}_{CS}$ , and ${\hat{a}}_{CL},\;SE_{\mathrm{req}}$ ( ${\hat{\beta }}_{CS})\;\mathrm{and}\;SE_{\mathrm{req}}$ ( ${\hat{a}}_{CL})\in$ {0.1 0.15}. For Aim 2b, $C_{0}\in \{$ 0.64, 0.72, 0.8} and for the difference, $d\in \left\{0.03,\;0.05\right\}$ .

#### Estimands/targets

(1) SEs of $\hat{C,}\;{\hat{\beta }}_{CS}$ and ${\hat{\alpha }}_{CL}$ ,

(2a) Sample sizes to attain a required SE, $SE_{\mathrm{req}},$ for $\hat{C},{\hat{\beta }}_{CS}$ and ${\hat{\alpha }}_{CL}$ (precision-based calculation),

(2b) Power and significance level when the estimated sample size, ${\hat{n}}_{\mathrm{req},\mathrm{app}\;}(C_{0},d)$ , is used to detect a difference d from a $C_{0}$ with power 1- β at a given significance level α (power-based calculation).

#### Methods

The estimated SEs, $SE_{\mathrm{app}}\left(\hat{C}\right),\;SE_{\mathrm{app}}({\hat{\beta }}_{CS})$ and $\;SE_{\mathrm{app}}\left({\hat{\alpha }}_{CL}\right)$ are obtained using formulae (9), (16) and (14), respectively. The alternative variance formulae (4), (12) and (13) for the C-statistic, calibration slope and calibration in the large, which require numerical integration, are also examined under DGM 3 and DGM 4. The corresponding estimated SEs are $SE_{\mathrm{app},\mathrm{NI}}\left(\hat{C}\right)$ , $SE_{\mathrm{app},\mathrm{NI}}({\hat{\beta }}_{CS})$ and $\;SE_{\mathrm{app},\mathrm{NI}}\left({\hat{\alpha }}_{CL}\right)$ , abbreviated to $SE_{\mathrm{NI}}\left(\hat{C}\right),\;SE_{\mathrm{NI}}({\hat{\beta }}_{CS})$ and $\;SE_{\mathrm{NI}}\left({\hat{\alpha }}_{CL}\right)$ . The estimated sample sizes for a precision-based calculation, ${\hat{n}}_{\mathrm{req},\;\mathrm{app}}(C)$ , ${\hat{n}}_{\mathrm{req},\;\mathrm{app}}\left(\beta_{CS}\right)$ and ${\hat{n}}_{\mathrm{req},\;\mathrm{app}}\left(\alpha_{CL}\right)$ , are obtained using formulae (17), (18) and (19), respectively. The estimated sample sizes for the alternative estimators are ${\hat{n}}_{\mathrm{req},\;\mathrm{NI}}\left(C\right)$ , ${\hat{n}}_{\mathrm{req},\;\mathrm{NI}}\left(\beta_{CS}\right)$ and ${\hat{n}}_{\mathrm{req},\;\mathrm{NI}}\left(\alpha_{CL}\right)$ . The estimated sample size for a power-based calculation, ${\hat{n}}_{\mathrm{req},\mathrm{app}}\left(C_{0},\;d\right),$ is obtained using formula (20).

#### Simulation process

For all simulations we used $n_{\mathrm{sim}}=10\text{,}000$ datasets.

Aim 1. We let θ denote a measure of predictive performance and ${\hat{\theta }}_{i}\;$ its estimate in the i th simulated dataset of size n.  The true SE, $SE_{\mathrm{true}}\left(\hat{\theta }\right),$ of $\hat{\theta }$ is approximated by the empirical SE, $SE_{\mathrm{emp}}\left(\hat{\theta }\right)=\sqrt{\frac{1}{n_{\mathrm{sim}}}\sum {\left({\hat{\theta }}_{i}-\bar{\theta }\right)}^{2}}$ , where $\bar{\theta }=\frac{1}{n_{\mathrm{sim}}}\sum {\hat{\theta }}_{i}.$ As $n_{\mathrm{sim}}$ is large, the empirical SEs can be regarded as the truth. The SE of $\hat{\theta }$ given by our estimators is obtained by plugging the values of n, p and C into our formulae and is denoted by $SE_{\mathrm{app}}\left(\hat{\theta }\right)$ and $SE_{\mathrm{NI}}\left(\hat{\theta }\right)$ .

Aim 2a. We let $n_{\mathrm{req}}(\theta )$ and $n_{\mathrm{req}}^{\left(e\right)}(\theta )$ denote the true required sample size and number of events, respectively, to attain a specified SE, $SE_{\mathrm{req}}\left(\hat{\theta }\right).$ This value is obtained after simulating a large number of datasets and hence can be regarded as the truth. The calculated sample size and number of events to obtain $SE_{\mathrm{req}}\left(\hat{\theta }\right)$ using our formulae are denoted by ${\hat{n}}_{\mathrm{req},\;\mathrm{app}}\left(\theta \right),\;{\hat{n}}_{\mathrm{req},\;\mathrm{NI}}\left(\theta \right)\;$ and ${\hat{n}}_{\mathrm{req},\mathrm{app}}^{\left(e\right)}(\theta )$ , ${\hat{n}}_{\mathrm{req},\mathrm{NI}}^{\left(e\right)}\left(\theta \right),$ respectively. If ${\hat{n}}_{\mathrm{req},\;\mathrm{app}}\left(\theta \right)$ and ${\hat{n}}_{\mathrm{req},\;\mathrm{NI}}\left(\theta \right)$ are close to $n_{\mathrm{req}}(\theta )$ , then our formulae for precision-based calculations performs well.

Aim 2b. We let ${\hat{n}}_{\mathrm{req},\mathrm{app}\;}(C_{0},d)\;$ and ${\hat{n}}_{\mathrm{req},\mathrm{app}}^{\left(e\right)}(C_{0},d)\;$ denote the calculated sample size and number of events, respectively, to detect a difference d at the significance level a=0.05 with power 1−β=0.9 . Datasets of size ${\hat{n}}_{\mathrm{req},\mathrm{app}\;}(C_{0},d)\;$ are simulated under the null and the alternative hypothesis. Without loss of generality, we assume that in the formulation of the null and alternative hypothesis, $C>\;C_{0}$ .

The probability of rejecting the null hypothesis when it is true is estimated by T $\mathrm{ype}\;I\;\mathrm{error}\left({\hat{n}}_{\mathrm{req},\mathrm{app}\;}\left(C_{0},d\right)\right)\;=\frac{1}{n_{\mathrm{sim}}}\;\sum I\left({\hat{C}}_{i,l}\geq C_{0}\right)\;$ where ${\hat{C}}_{i,l}={\hat{C}}_{i}-z_{1-a}\times \;\sqrt{{\hat{\mathrm{var}}}_{\mathrm{DL}}\left({\hat{C}}_{i}\right)}$ and ${\hat{\mathrm{var}}}_{\mathrm{DL}}({\hat{C}}_{i})$ denotes the estimated variance using DeLong’s formula (3).

The probability of rejecting the null hypothesis when the alternative is true is estimated by

$$\mathrm{Power}\left({\hat{n}}_{\mathrm{req},\mathrm{app}\;}\left(C_{0},d\right)\right)\;=\frac{1}{n_{\mathrm{sim}}}\;\sum I\left({\hat{C}}_{i,l}\leq C_{0}\right).$$

For large $n_{\mathrm{sim}}$ , these approximations can be treated as the rejection probabilities. A T $\mathrm{ype}\;I\;\mathrm{error}\left({\hat{n}}_{\mathrm{req},\mathrm{app}\;}\left(C_{0},d\right)\right)$ that is close to 0.05 and a $\mathrm{Power}\left({\hat{n}}_{\mathrm{req},\;\mathrm{app}\;}\left(C_{0},d\right)\right)$ that is close to 0.9 are suggestive of good performance from our formula for power-based calculations.

#### Performance measures

(1) Percentage bias in the estimated SE of $\hat{\theta }$ for a given number of events:

$$\%\;\mathrm{Bias}\left(SE_{\mathrm{app}}\left(\hat{\theta }\right)\right)=100\;\left(\;\frac{{\mathrm{SE}}_{\mathrm{app}}\left(\hat{\theta }\right)}{SE_{\mathrm{true}}\left(\hat{\theta }\right)}\;-1\right)$$

(2a) Percentage bias in the estimated sample size for a specified SE:

$$\%\;\mathrm{Bias}\left({\hat{n}}_{\mathrm{req},\mathrm{app}}(\theta )\right)=100\left(\frac{{\hat{n}}_{\mathrm{req},\;\mathrm{app}}\left(\theta \right)}{n_{\mathrm{req}}\left(\theta \right)}-1\right)$$

(2b) Type-1 error rate and power when a sample of size ${\hat{n}}_{\mathrm{req},\mathrm{app}\;}(C_{0},d)\;$ is used to detect a difference d from $C_{0}$ with a given power and significance level.

### 5.2 Results

For DGM 1 and 3, we assessed both aims, while for DGMs 2 and 4 we primarily focus on Aim 1. For DGM 3, we present main results in Tables 1 and 2 for p∈{10%, 30%}, C  up to 0.85, number of events up to 200 , $SE_{\mathrm{req}}\left(\hat{C}\;\right)\in$ {0.0125, 0.025} and $SE_{\mathrm{req}}$ ( ${\hat{\beta }}_{CS})\;\mathrm{and}\;SE_{\mathrm{req}}$ ( ${\hat{\alpha }}_{CS})\in$ {0.1 0.15}. Full simulation results can be found in Supplementary Material 2 which also includes results for DGMs 1, 2 and 4.

**Table 1.** DGM 3. % Bias of the estimated standard errors for $\hat{C},\;{\hat{\beta }}_{cs}$ and ${\hat{\alpha }}_{CL},$ calculated over 10,000 simulations for true prevalence values 10% and 30% and true C-statistic of 0.64, 0.72, 0.8 and 0.85.

			C-statistic			Calibration slope			Calibration in the large		
p	C	$n_{e}$	$SE_{\mathrm{true}}$	%Bias $SE_{\mathrm{app}}$	%Bias $SE_{\mathrm{NI}}$	$SE_{\mathrm{true}}$	%Bias $SE_{\mathrm{app}}$	%Bias $SE_{\mathrm{NI}}$	$SE_{\mathrm{true}}$	%Bias $SE_{\mathrm{app}}$	%Bias $SE_{\mathrm{NI}}$
0.1	0.64	50	0.041	−3	−3	0.295	−2	−1	0.145	1	1
		100	0.028	0	0	0.205	0	0	0.104	−1	−1
		200	0.020	0	0	0.147	−1	−1	0.074	−2	−2
0.1	0.72	50	0.036	0	0	0.188	−3	−1	0.148	0	0
		100	0.026	1	0	0.134	−4	−2	0.106	−1	−1
		200	0.018	0	1	0.093	−2	0	0.074	0	0
0.1	0.8	50	0.032	0	0	0.142	−8	−1	0.155	−1	−1
		100	0.022	0	−1	0.100	−7	0	0.109	0	0
		200	0.016	0	−1	0.069	−6	1	0.076	0	0
0.1	0.85	50	0.027	1	0	0.126	−13	−1	0.160	1	0
		100	0.019	2	−1	0.088	−12	1	0.113	1	0
		200	0.014	2	0	0.062	−11	1	0.079	2	1
0.3	0.64	50	0.041	−4	−4	0.305	−1	−1	0.154	−2	−2
		100	0.029	−1	0	0.215	−1	−1	0.107	−1	0
		200	0.020	0	0	0.151	0	0	0.076	−1	−1
0.3	0.72	50	0.037	−1	−1	0.200	−4	−1	0.158	−4	−3
		100	0.027	0	0	0.142	−5	−3	0.108	−1	1
		200	0.019	−1	−1	0.098	−2	1	0.077	−2	0
0.3	0.8	50	0.033	−1	−2	0.157	−9	−3	0.163	−5	−1
		100	0.023	0	−1	0.108	−7	0	0.115	−6	−1
		200	0.016	0	−1	0.076	−7	0	0.080	−4	1
0.3	0.85	50	0.028	0	0	0.141	−14	−3	0.172	−10	−2
		100	0.020	0	−1	0.098	−13	−1	0.121	−9	−1
		200	0.014	1	0	0.067	−10	2	0.085	−8	0

**Table 2.** DGM 3. Number of events for a specified standard error for $\hat{C}$ , ${\hat{\beta }}_{cs}$ and ${\hat{\alpha }}_{CL}.$

		C-statistic				Calibration slope				Calibration in the large		
p	C	$SE_{\mathrm{req}}$	$n_{\mathrm{req}}^{\left(e\right)}$	%Bias ${\hat{n}}_{\mathrm{req},\mathrm{app}}$	$\%\mathrm{Bias}{\hat{n}}_{\mathrm{req},\mathrm{NI}}$	$SE_{\mathrm{req}}{\hat{\beta }}_{CS}\;\&{\;\hat{\alpha }}_{CL}$	$n_{\mathrm{req}}^{\left(e\right)}$	$\%\mathrm{Bias}{\hat{n}}_{\mathrm{req},\mathrm{app}}$	$\%\mathrm{Bias}{\hat{n}}_{\mathrm{req},\mathrm{NI}}$	$n_{\mathrm{req}}^{\left(e\right)}$	$\%\mathrm{Bias}{\hat{n}}_{\mathrm{req},\mathrm{app}}$	$\%\mathrm{Bias}{\hat{n}}_{\mathrm{req},\mathrm{NI}}$
0.10	0.64	0.0125	541	−1	0	0.1	453	0	1	115	−1	−1
0.10	0.72	0.0125	447	1	2	0.1	198	−6	−2	120	−3	0
0.10	0.80	0.0125	329	3	1	0.1	118	−15	−4	130	−9	1
0.10	0.85	0.0125	241	4	−2	0.1	94	−23	−4	146	−17	−3
0.30	0.64	0.0125	695	−1	0	0.1	625	0	−1	153	−1	−1
0.30	0.72	0.0125	586	−1	−1	0.1	282	−2	−2	166	−1	0
0.30	0.80	0.0125	420	3	2	0.1	180	−9	−4	192	−5	0
0.30	0.85	0.0125	323	1	−1	0.1	149	−14	−2	216	−15	0
0.10	0.64	0.025	136	−1	0	0.15	209	−5	−3	52	−2	−2
0.10	0.72	0.025	112	1	−1	0.15	89	−7	−4	55	−5	−3
0.10	0.80	0.025	86	−1	1	0.15	54	−17	−7	58	−9	0
0.10	0.85	0.025	62	2	−3	0.15	44	−27	−9	65	−17	−3
0.30	0.64	0.025	173	−1	0	0.15	282	−1	−2	70	−3	−4
0.30	0.72	0.025	144	1	0	0.15	125	−2	−1	76	−3	−3
0.30	0.80	0.025	110	−2	−2	0.15	81	−10	−5	86	−6	−1
0.30	0.85	0.025	81	0	−2	0.15	70	−19	−8	97	−15	−1

*Note*. % Bias of the estimated sample size (and number of events), calculated over 10,000 simulations for true prevalence values 10% and 30% and true C-statistic of 0.64, 0.72, 0.8 and 0.85. ${\hat{n}}_{\mathrm{req}}^{\left(e\right)}$ denotes the required number of events.

#### DGM 1: Conditional normal linear predictor with equal variances

For the estimated C-statistic, $SE_{\mathrm{app}}\left(\hat{C}\right),\;$ there was in good agreement with $SE_{\mathrm{true}}\left(\hat{C}\right)\;$ across all model strength, prevalence and number of events scenarios (Table S1). The largest bias was less than 4% in absolute value. Similarly, ${\hat{n}}_{\mathrm{req},\;\mathrm{app}}(C)$ was very close to $n_{\mathrm{req}}(C)$ for all values of $p,\;C\;\mathrm{and}\;SE_{\mathrm{req}}$ for $\hat{C.}$ The power and Type I error for the estimated sample size were very close to the nominal values of 90% and 5%, respectively, for values of $C_{0}$ up to 0.8. (Table S3).

For the estimated calibration slope, the SEs were estimated well for values of C up to 0.8 but tended to be underestimated for higher values of C (Table S1) . The worst bias for values of C up to 0.8 was –8%, and for C=0.9 , it worsened to –20%. The deterioration in the performance of our formula for the variance of ${\hat{\beta }}_{CS}\;$ was expected for very high values of C and was due to the higher efficiency of the LDA estimator compared to logistic regression for high values of C. This was confirmed by comparing the efficiency of logistic regression against LDA for a range of values for p and C , when data were generated under DGM 1 (see Figure S1 of Supplementary Material 2). The estimated number of events required to achieve a specified SE was underestimated by a factor of at most 15% for values of C up to 0.8 and deteriorated further to 37% for C = 0.9 (Table S2).

#### DGM 2: Conditional Normal linear predictor with unequal variances

Results for the estimated SEs of ${\hat{\beta }}_{CS}$ were very similar to those for DGM 1 for C up to 0.8. For values of C≥0.85 , the bias in the estimation of the SE of ${\hat{\beta }}_{CS}$ was increased by at most 4% compared to the bias observed in DGM 1. These results are presented in Table S4 of Supplementary Material 2.

#### DGM 3: Marginally Normal linear predictor

For the estimated C-statistic and calibration slope, the results were very similar to the results seen for DGM 1 and DGM 2. Arguably, the similarity is due to the fact that when the marginal distribution of the linear predictor is Normal, the conditional distribution of the linear predictor given the outcome is also approximately Normal, with the corresponding variances in the cases and control groups being very similar for values of C up to 0.8 (see Figure S3 in Supplementary Material 1).

For $\hat{C}$ , the SEs from our closed-form formulae were estimated very well for all values of C and prevalence (Table 1 and S5). The largest bias was 4% in absolute value and it occurred for C=0.64.

The results from using formula (4) that requires numerical integration were very similar, with the highest bias being less than 4%. This amount of bias occurred only for $n_{e}=50$ and therefore is likely to be due to small-sample bias. Similarly, ${\hat{n}}_{\mathrm{req},\;\mathrm{app}}(C)$ and ${\hat{n}}_{\mathrm{req},\;\mathrm{NI}}(C)$ were very close to $n_{\mathrm{req}}(C)$ for all values of $SE_{\mathrm{req}}$ ( $\hat{C}$ ) , p and C (Table 2 and S6). The power and Type I error for the estimated sample size were very close to the nominal values of 90% and 5%, respectively, for values of $C_{0}$ up to 0.8. (Table S7).

For the estimated calibration slope, the SEs from our closed-form formula were estimated well for values of C up to 0.8 but tended to be underestimated for higher values of C (Tables 1 and S5) . The worst bias for values of C up to 0.8 was –10%, and for C=0.9 , it worsened to –22%. The estimated number of events required to achieve a specified SE was underestimated by a factor of at most 20% for values of C up to 0.8 and deteriorated further to 40% for C=0.9 (Table 2 and S6). The results for the calibration in the large were similar to those seen for calibration slope. The SEs from our closed-form formula were estimated well for values of for C up to 0.8, with a maximum bias of 8%, but deteriorated for higher values of C , with a maximum bias of –22% when C = 0.9 (Tables 1 and S5). A similar pattern was observed for the number of events required to achieve a specified SE (Tables 2 and S6).

The performance of the variance estimators that require the use of numerical integration was very good with minimal bias across most scenarios under DGM 3 (see Tables 1, 2 and S5, S6). The maximum bias (in absolute value) for the SE of the calibration in the large was 3%. For calibration slope, the maximum bias was –4%, except when the number of events was small (50) in which case the maximum bias was –8%. For values of C>0.8 , a range of values for which the closed-form formulae performed less well, the variance estimators that require the use of numerical integration should be used instead.

For $n_{\mathrm{sim}}=10\text{,}000$ , the maximum Monte Carlo Standard Error (MCE) for the empirical SEs $SE_{\mathrm{true}}\left(\hat{C}\right)$ , $SE_{\mathrm{true}}({\hat{\beta }}_{CS})$ and $SE_{\mathrm{true}}({\hat{\alpha }}_{CL})$ were 0.0005, 0.0036 and 0.0023, respectively. These were the magnitudes of MCE also for DGMs 1, 2 and 4.

#### DGM 4: Marginally skewed linear predictor

The SE of $\hat{C}$ was estimated well for values of C up to 0.8, but it was underestimated for higher values of C (Table S8). The underestimation was more pronounced for lower values of prevalence. In particular, the SE of $\hat{C}$ was underestimated by a factor of up to 8% for C up to 0.8 and by a factor of up to 17% when C was 0.9. For the calibration slope, the opposite pattern was observed, with the true SE of ${\hat{\beta }}_{CS}\;$ being overestimated by our closed-form formula for low C and underestimated for high C. For values of C up to 0.8, the SE was overestimated by up to a factor of 13%, with the highest overestimation occurring when C=0.64. The worst underestimation of 17% occurred when C=0.9. For calibration in the large, the SE of ${\hat{\alpha }}_{CL}$ was estimated well by our close-form formula for all values of C when p = 0.05 , but for other prevalence values, it was underestimated by a factor of up to 27% when C>0.8 .

#### Summary

To summarise, our simulations suggest that our closed-form sample size formulae based on the C-statistic, the calibration slope and the calibration in the large estimate well the required sample size for values of C at least up to 0.8 , regardless of the distribution of the linear predictor and the outcome prevalence. More precisely, we have seen that under the assumption of marginal normality for the linear predictor, the closed-form formula for precision-based calculation based on C worked very well across all considered values of C and p. The results obtained by the formula that uses numerical integration were very similar. For calibration slope and calibration in the large, the corresponding closed-form formulae worked well for values of C up to 0.8 ; for higher values of C , the variance estimators which require the use of numerical integration should be used.

## 6 Survival outcomes and further considerations

In Sections 3 to 5, we focused on risk-prediction models for binary outcomes. However, in health research, outcomes are often time-to-event (also known as Survival Outcomes, e.g. time to death, time to relapse), and our formulae are not designed to apply to these settings. Expressing the variance of Harrell’s C-index,^25^ which is the most popular concordance measure, and the variance of the calibration slope for survival outcomes in a form that does not depend on patient-level information is cumbersome and may not result in simple formulae analogous to those for binary outcomes.

We next discuss two simple approaches for variance and sample size estimation that can be used in specific scenarios for risk models with survival outcomes. In the first, we make use of our variance and sample size formulae for binary outcomes and apply them to survival outcomes. In the second, we assume that an estimate of the variance of the estimated performance measures is available from a previous validation exercise. Using the fact that the asymptotic variance is proportional to the sample size and using a variance estimate from an existing study, we then obtain formulae to estimate the sample size to attain a required variance for the estimated C-index/C-statistic and the estimated calibration slope.

### 6.1 Use of variance estimators for binary outcomes in survival-data settings

As the regression parameters from logistic and Cox regression are similar when the probability of event occurrence is low,^26,27^ we investigated whether our formulae for the variances of $\hat{C}$ and $\;{\hat{\beta }}_{cs}$ hold for survival data settings by using the proportion of events (observed failures), $p_{e}$ , as prevalence and replacing the true value of the C -statistic in our variance formulae for binary outcomes by Harrell’s C-index, denoted by $C_{H}$ . Simulation studies for survival outcomes analogous to the ones in Section 5 were carried out to address Aim 1 under a modified DGM where survival outcomes were generated from a proportional hazards regression model. The marginal distribution of the linear predictor was Normal, and its parameters were varied to achieve a range of desired values for $C_{H}\in \{0.64,\;0.72,\;0.8,\;0.85,\;0.9\}\;$ and proportion of events, $p_{e}\in \left\{0.05,\;0.1,\;0.2\right\}.$ The censoring mechanism was chosen to correspond to random censoring, where individuals who have not experienced the event may have different censoring times, but we assume that the time of censoring is unrelated to the unobserved survival time (non-informative censoring). Details of the DGM and results are presented in the Supplementary Material 2.

The variance formulae for binary data performed best when the proportion of failures was small, i.e. the proportion of censored individuals was very high (Table S9). For the C-index, when $p_{e}=0.05$ , the SEs of ${\hat{C}}_{H}$ obtained from our formula for binary outcomes were lower than the true SEs by a factor of –13% to –2%, with the worse bias corresponding to lower values of $C_{H}$ . For $p_{e}=0.1$ , the true SEs were underestimated by a factor of –10% to 1% when $C_{H}\leq 0.85$ and overestimated by a factor of up to 7% when $C_{H}=0.9$ . When $p_{e}=0.2$ , the overestimation for high $C_{H}\;$ became more pronounced, with a factor of up to 18%. This pattern can be explained by the fact that the formula for binary data considers only the proportion of events or non-events (whichever is the lowest) and not the actual survival times. So, as censoring decreases and the prevalence approaches one, even though there is a lot of information in the data with the majority of the survival times being observed, the amount of information used in the formula for binary data is compromised by only considering the number of non-events. Hence, the SEs tend to be larger than the true values as prevalence increases (and censoring decreases). For calibration slope, the SEs of ${\hat{\beta }}_{cs}$ obtained from our formula for binary outcome of Section 3.2 were close to the true SEs for values of $p_{e}\;\mathrm{up}\;\mathrm{to}\;0.1.$ In particular, when $p_{e}=0.05$ and 0.1, the highest bias was –6% and 11% respectively, but it increased to 24% for $p_{e}=0.2$ .

In summary, when dealing with survival data, our formula for the variance of ${\hat{\beta }}_{cs}$ that treats the outcomes as binary worked reasonably well when the prevalence was low ( $p_{e}\leq \;$ *0.1*). Hence, the corresponding formula for sample size estimation based on the calibration slope can be used although it will slightly overestimate the sample size, thus providing a conservative sample size estimation. The sample size formula based on the C-statistic can also be used for $p_{e}\leq 0.1$ with caution as it may underestimate the sample size.

### 6.2 Sample size calculation when estimates for measures of predictive performance are available from a previous validation study

We also considered an alternative approach to sample size calculation which requires an existing validation dataset called the ‘reference dataset’ and an estimate of θ and its variance from that dataset.

We let ${\mathrm{var}}_{\mathrm{asymp}}\left(\hat{\theta }\right)$ denote the asymptotic variance of θ and ${\mathrm{var}}_{n}\left(\hat{\theta }\right)$ the true variance of $\hat{\theta }$ for a dataset of size n . By definition, ${\mathrm{var}}_{\mathrm{asymp}}\left(\hat{\theta }\right)=n\times \underset{n\to \infty }{\lim}{\mathrm{var}}_{n}\left(\hat{\theta }\right)$ . Assuming that a reference validation dataset of size $n^{*}$ is available, then the asymptotic variance of θ can be approximated by ${\mathrm{var}}_{\mathrm{app},\mathrm{asymp}}\left(\hat{\theta }\right)\;=n^{*}\times {\hat{\mathrm{var}}}_{n^{*}}\left(\hat{\theta }\right)$ , where ${\hat{\mathrm{var}}}_{n^{*}}\left(\hat{\theta }\right)$ denotes the estimated variance of $\hat{\theta }$ in the reference dataset. For example, for binary outcomes ${\hat{\mathrm{var}}}_{n^{*}}\left(\hat{C}\right)$ is calculated using DeLong’s formula (3). For a new dataset of size n

(21) $${\mathrm{var}}_{n}\left(\hat{\theta }\right)=\frac{{\mathrm{var}}_{\mathrm{app},\mathrm{asymp}}\left(\hat{\theta }\right)}{n}=\frac{n^{*}}{n}\;{\hat{\mathrm{var}}}_{n^{*}}\left(\hat{\theta }\right)\;\;$$

In practice, the larger the size of the reference dataset, the better ${\mathrm{var}}_{\mathrm{asymp}}\left(\hat{\theta }\right)$ will be approximated by ${\mathrm{var}}_{\mathrm{app},\mathrm{asymp}}\;\left(\hat{\theta }\right).\;$ For sample size calculations, if an estimate of the variance, $\;{\hat{\mathrm{var}}}_{n^{*}}\left(\hat{\theta }\right),$ of $\hat{\theta }$ is available from a reasonably sized reference dataset of size $n^{*}$ , then solving equation (21) for n, the sample size required to estimate θ with the required variance ${\mathrm{var}}_{\mathrm{req}}\left(\hat{\theta }\right)$ is

(22) $${\hat{n}}_{\mathrm{req}}\left(\hat{\theta }\right)=\frac{n^{*}\;{\hat{\mathrm{var}}}_{n^{*}}\;\left(\hat{\theta }\right)\;}{\mathrm{va}r_{\mathrm{req}}\left(\hat{\theta }\right)}\;$$

It is noted that (22) assumes that the outcome prevalence in the newly collected data is the same as the prevalence in the reference dataset.

## 7 Real data illustration

A risk model was developed^28^ to predict the risk of in-hospital mortality for patients undergoing heart valve surgery based on pre-operative patient characteristics. Heart valve surgery has an associated in-hospital mortality rate of 4% to 8%. The development sample consisted of 16,679 patients in Great Britain and Ireland who had surgery between 1995 and 1999, and the proportion of deaths was 6.4%. The risk model included 13 categorical and continuous predictors. The model was validated in a sample of 16,160 patients who had surgery in the five following years. The proportion of deaths in the validation sample was 5.7%. The estimated C-statistic was 0.77, and the calibration slope 1.00. The estimated calibration in the large was not available. Using the individual-level data for all patients in the validation data, the De Long’s estimate of the SE of $\hat{C}$ was 0.00765, and the model-based estimate of the SE of ${\hat{\beta }}_{CS}$ was 0.0349.

Suppose we wish to collect new data and assess the performance of this model in a contemporary patient population. We perform sample size calculations using:

- the formulae of Section 4 for precision- and power-based calculations and
- the formula of Section 6.2 which requires the presence of previous validation data.

We compare the recommendations based on the approaches above with the current guideline recommendation of at least 100–200 events. The code for the sample size calculations that follow can be found in Supplementary Material 3.

### Sample size calculation based on anticipated values for the outcome prevalence and C-statistic

#### Precision-based sample size calculation

Based on information available from the literature, the discriminatory ability of the model is reflected by an anticipated population value of C=0.77 and an anticipated outcome prevalence of 5.7%. Suppose we require C to be estimated with an SE of 0.025 and the calibration slope and calibration in the large with an SE of 0.15, so ${\mathrm{var}}_{\mathrm{req}}\left(\hat{C}\right)=0.025^{2}$ and ${\mathrm{var}}_{\mathrm{req}}({\hat{\beta }}_{CS})={\mathrm{var}}_{\mathrm{req}}\left({\hat{\alpha }}_{CL}\right)=0.15^{2}$ . Using the formulae that require numerical integration, the required number of patients (events) are 1610(92), 940(54) and 900(52) based on the C-statistic, the calibration slope and the calibration in the large, respectively (number of patients is rounded up to the nearest 10). So, the recommended size would be 1610 patients (92 events). The corresponding number of patients (events) from the closed-form formulae are very similar overall, 1600(92), 850(49) and 890(51) based on the C-statistic, the calibration slope and the calibration in the large, respectively.

#### Power-based sample size calculation

Suppose that the risk model is considered to be outdated and it is hypothesized that its discriminatory ability is now lower. Suppose we wish to collect enough data to be able to detect a difference of 0.05 from the null value of C with power 90% at the 5% significance level, where $C_{0}=0.77$ and $C_{1}=0.72$ . Based on a one-sided test, using formula (20), the required sample size (number of events) is 3690 (211).

### Sample size calculation based on existing estimates for the measures of predictive performance from an existing validation study

The reference dataset is considered to be the existing validation dataset of 16,160 patients with ${\mathrm{var}}_{16160}\left(\hat{C}\right)=0.00765$ and ${\mathrm{var}}_{16160}\;({\hat{\beta }}_{CS})=0.0349.$ Using formula (22), the estimated sample sizes (events) to obtain ${\mathrm{var}}_{\mathrm{req}}\left(\hat{C}\right)=0.025$ and ${\mathrm{var}}_{\mathrm{req}}({\hat{\beta }}_{CS})=0.15$ were 1520 (87) and 850(49), close to the sizes recommended by the use of our formulae.

### Current guideline recommendations of 100 events

Given an assumed outcome-prevalence of 5.7%, a validation sample of at least 1760 patients would be required to ensure at least 100 events are observed. This size would correspond to an SE $\left(\hat{C}\right)$ = 0.024, SE $\left({\hat{\beta }}_{CS}\right)$ = 0.104 and SE $\left({\hat{\alpha }}_{CL}\right)$ = 0.106. Also, it would allow the detection of a difference of 0.074 from $C_{0}=$ 0.77 with power 90% at the 5% significance level.

## 8 Discussion

In recent years, sample size estimators for the development of risk models for continuous, binary and survival outcomes^12,29^ and for the external validation of risk models for continuous outcome^30^ have been suggested. In this work, we propose sample size estimators for the validation of risk models for binary outcomes, which fill an important gap in the literature, and will enable researchers to make quick and informed sample size choices when designing their validation studies. Also, when it is only feasible to collect limited data due to cost, time or other restrictions, our estimators may inform researchers about the anticipated precision of the estimated validation measures or the power with which a desired difference can be detected.

Analogous calculations can be performed using simulation,^8,10,11^ akin to the approaches used in this paper to obtain the true values of SEs and sample sizes under certain assumptions. Simulation will typically require more programming knowledge compared to applying our formulae, and it will be more accurate, although our estimators have shown very good performance in a wide range of scenarios. Thus, simulation remains an alternative useful tool, particularly when it is required to perform sample size calculations tailored to the characteristics of a particular study.

The decision about the required sample size in validation studies for binary outcomes has so far been predominantly based on the recommendation of at least 100 events (or non-events). This recommendation partly accounts for outcome prevalence but does not take into account the model strength, as reflected by the C-statistic. So, for a given prevalence, the recommended sample size will be fixed even though different model strengths would correspond to different precisions for the estimates of the performance measures. It also does not differentiate between precision- and power-based sample size requirements.

We have proposed easy-to-use formulae for sample size calculations for external validation studies, based on the C-statistic, the calibration slope and calibration in the large which are standard measures for the predictive performance of a risk model for binary outcomes. In particular, we have proposed formulae to estimate the sample size required to ensure either that: (a) the true variance of an estimated measure of predictive performance is approximately equal to a specified value (precision-based calculation) or (b) there is sufficient power to detect a difference in the estimate of a measure of predictive performance from a target value (power-based calculation). To achieve this, we derived formulae for the variance of the estimated performance measures as a function of sample size and the true values of the C-statistic and outcome prevalence under the assumption of conditional normality given the outcome for the C-statistic and calibration slope and marginal normality for the calibration in the large.

### Assessing departure from model assumptions

We used simulation to assess the validity of our variance and sample size formulae when the assumptions about the distribution of the linear predictor were met and under reasonable departures from these assumptions. We have found that under the assumption of marginal normality (DGM 3), our closed-form variance formula for the C-statistic performed well across a range of values range of values for the true C (0.64,0.72,0.80, 0.85,0.9) and true prevalence (5%, 10%, 30%). Under marginal normality, our closed-form formulae for the calibration slope and calibration in the large performed well for values of C up to 0.8 but performed less well for C>0.8 . Therefore, for C>0.8 , we suggest that the estimators that require the use of numerical integration be used.

When the linear predictor was severely skewed, both marginally and conditionally for the cases and the controls, our formulae performed well for values of C  up to 0.8, but their performance tended to deteriorate for higher values of C. Non-normality is more likely to be a concern in small models with mostly categorical predictors. The scenario assessed in DGM 4 included only binary predictors, resulting in a highly skewed conditional distribution for the linear predictor. In practice, risk models most often include a number of weakly correlated predictors, and unless this number is very small or there are only binary predictors with extreme prevalences, the distribution of the linear predictor is likely to be approximately marginally Normal, a scenario in which our variance and sample size formulae have been seen to perform well. Importantly, marginal normality also corresponds to approximate conditional normality of the linear predictor with similar variances for cases and controls as long as C is not too high ( C≤0.85 ), a condition required by our closed-form variance formulae for the C-statistic and calibration slope. Consequently, our respective formulae for variance and sample size are expected to be valid in these settings. Nevertheless, if there are concerns about non-normality of the linear predictor and the anticipated C is high, the approach that involves numerical integration may also be considered, although this will require additional information from the user regarding the shape of the assumed distribution.

### Selection of the required SE for precision-based and the acceptable difference for power-based sample size calculations

Both precision- and power-based calculations using our formulae require the input of values for the C-statistic and the outcome prevalence. Anticipated values for these quantities can be obtained from previous development and/or validation studies or expert clinical opinion.

If we were to adhere to the existing rule of 100 events, we would obtain, approximately, $SE(\hat{C})$ between 0.02 and 0.04, $SE({\hat{\beta }}_{CS})$ between 0.1 and 0.3, and $SE\left({\hat{\alpha }}_{CL}\right)$ between 0.1 and 0.18, depending on the prevalence and the C-statistic (Figure 1). For moderate values of C (0.7–0.8) and small prevalence (0.05–0.2), $SE(\hat{C})$ is between 0.025 and 0.03, $SE({\hat{\beta }}_{CS})$ between 0.1 and 0.17 and $SE\left({\hat{\alpha }}_{CL}\right)$ between 0.10 and 0.13.

Our formulae for precision-based sample size calculations additionally require the researcher to provide a value for the required SE of the estimated performance measure. The decision regarding the required precision for $\hat{C}$ is subjective and could depend on the specific validation study. The cut-off points of 0.6, 0.7 and 0.8 for the C-statistic have been referred to as the thresholds for low, medium and high model strength.^31^ Hence, a reasonable level of precision could be reflected by a maximum SE of 0.025, which would ensure that the 95% confidence interval of approximate width of 0.10 does not cross more than one cut-off points. To achieve this SE, a validation study would require between 60 and 170 events if C is between 0.64 and 0.85 and p is between 0.05 and 0.3. For the calibration slope and calibration in the large, achieving an SE of 0.15 would require 40–280 and 50–100 events, respectively, for the same range of values for C and p .

Our formulae for power-based calculations additionally require the specification of a value for the difference from the target value of C . For example, assuming that the outcome prevalence is 10%, and the target value of C is $C_{0}=$ 0.72 with $C_{1}=0.75,$ 470 events are required to detect a difference of 0.03 with power 80% at 95% significance level, a number that may not be realistically attainable in many clinical settings.

### Sample size calculations for survival outcomes

Our formulae for the sample size calculations were developed for validation studies with binary outcomes. We have also investigated the validity of these formulae for survival data, where prevalence is taken to be the proportion of observed failures. The formulae for calibration slope can be applied when prevalence is 10% or less and provide a slightly conservative sample size recommendation. The formulae for the C-statistic should be used with caution when p≤10% as this may lead to underestimation of the required sample size. Alternatively, provided that an estimate of the variance of a measure of predictive performance is available from an existing validation study, the sample size for a precision-based calculation can be calculated by exploiting the relationship between the asymptotic variance and sample size.

## Supplemental Material

sj-pdf-1-smm-10.1177_09622802211007522 - Supplemental material for Estimation of required sample size for external validation of risk models for binary outcomes Supplemental material, sj-pdf-1-smm-10.1177_09622802211007522 for Estimation of required sample size for external validation of risk models for binary outcomes by Menelaos Pavlou, Chen Qu, Rumana Z Omar, Shaun R Seaman, Ewout W Steyerberg, Ian R White and Gareth Ambler in Statistical Methods in Medical Research

sj-pdf-2-smm-10.1177_09622802211007522 - Supplemental material for Estimation of required sample size for external validation of risk models for binary outcomes Supplemental material, sj-pdf-2-smm-10.1177_09622802211007522 for Estimation of required sample size for external validation of risk models for binary outcomes by Menelaos Pavlou, Chen Qu, Rumana Z Omar, Shaun R Seaman, Ewout W Steyerberg, Ian R White and Gareth Ambler in Statistical Methods in Medical Research

sj-txt-3-smm-10.1177_09622802211007522 - Supplemental material for Estimation of required sample size for external validation of risk models for binary outcomes Supplemental material, sj-txt-3-smm-10.1177_09622802211007522 for Estimation of required sample size for external validation of risk models for binary outcomes by Menelaos Pavlou, Chen Qu, Rumana Z Omar, Shaun R Seaman, Ewout W Steyerberg, Ian R White and Gareth Ambler in Statistical Methods in Medical Research
