# Supplementary material for: Estimation of required sample size for external validation of risk models for binary outcomes
Source: Stat Methods Med Res. 2021 Apr 21;30(10):2187–206. doi: 10.1177/09622802211007522 (PMC8529102; doi:10.1177/09622802211007522)
Supplement: sj-pdf-1-smm-10.1177_09622802211007522 - Supplemental material for Estimation of required sample size for external validation of risk models for binary outcomes [file sj-pdf-1-smm-10.1177_09622802211007522.pdf]

Supplementary Material 1

Let Y denote the binary outcome for a given patient, $p = Pr(Y = 1)$ the outcome prevalence and n the total number of subjects in the validation dataset. The linear predictor, i.e. the predicted log-odds, is denoted by η. Let G_0 denote the group of n_0 subjects with $Y = 0$ (controls) and G_1 the group of n_1 subjects with $Y = 1$ (cases). In this supplement we provide proofs for the formulae for the variances of the estimated C-statistic, the calibration slope and calibration in the large.

1 Proof of the formulae for the variance of the estimated C-statistic

Let $\eta_i^{(1)}$, $i = 1, \ldots, n_0$, and $\eta_j^{(0)}$, $j = 1, \ldots, n_1$ denote the linear predictor for the ith case and jth control, respectively. Also, $\boldsymbol{\eta}^{(0)} = \left(\eta_1^{(0)}, ..., \eta_{n_0}^{(0)}\right)^T$, $\boldsymbol{\eta}^{(1)} = \left(\eta_1^{(1)}, ..., \eta_{n_1}^{(1)}\right)^T$ and $\boldsymbol{\eta} = \left(\boldsymbol{\eta}^{(0)T}, \boldsymbol{\eta}^{(1)T}\right)^T$.
The Mann-Whitney estimator of C, the probability that a randomly selected observation from the sample represented by G_0 will be less than or equal to a randomly selected observation from the population represented by G_1, is:

$$\hat{C} = \frac{1}{n_0 n_1} \sum_i^{n_0} \sum_j^{n_1} I(\eta_i^{(1)}, \eta_j^{(0)}) \tag{1}$$

We define for each case, i, and each control, j, the quantities

$$V_{10}(\eta_i^{(1)}) = V_{10,i} = \frac{1}{n_0} \sum_{j=1}^{n_0} I(\eta_i^{(1)}, \eta_j^{(0)}) \text{ and}$$
$$V_{01}(\eta_j^{(0)}) = V_{01,j} = \frac{1}{n_1} \sum_{i=1}^{n_1} I(\eta_i^{(1)}, \eta_j^{(0)})$$

Therefore equation (1) can also be written as $\sum_{i=1}^{n_1} V_{10,i}/n_1$ or $\sum_{j=1}^{n_0} V_{01,j}/n_0$.
The DeLong's estimator for the variance of $\hat{C}$ is:

$$\widehat{\text{var}}(\hat{C}) = \frac{S_{10}}{n_1} + \frac{S_{01}}{n_0} + \frac{S_{11}}{n_1 n_0}, \tag{2}$$

where

$$S_{10} = \frac{1}{n_1 - 1} \sum_i \left(V_{10,i} - \hat{C}\right)^2 \tag{3}$$
$$S_{01} = \frac{1}{n_0 - 1} \sum_j \left(V_{01,j} - \hat{C}\right)^2 \tag{4}$$
$$S_{11} = \sum_i \sum_j \left(I(\eta_i^{(1)}, \eta_j^{(0)}) - \hat{C}\right)^2$$

As Delong (1988) and Cleve (2012) do, we subsequently omit the third term on the right-hand side of equation (2) as it is negligible when n_0 and n_1 are large. We first obtain an expression for the variance of the C-statistic that is based on the asymptotic variance based on DeLong's expression. Subsequently we aim to obtain a closed-form expression for the variance of the estimated C-statistic that is free from patient-level information. To achieve this we make the assumption that the distribution of the linear predictor is conditionally Normal given the binary outcome.

Taking the expectation of (2) we have,

$$E(\widehat{\text{var}}(\hat{C})) = \frac{E(S_{10})}{n_1} + \frac{E(S_{01})}{n_0} \tag{5}$$

Replacing $n_1 - 1$ and $n_0 - 1$ by n_1 and n_0 when they are relatively large, in (3) and (4), respectively

$$\begin{aligned}
\frac{E(S_{10})}{n_1} =& \frac{1}{n_1^2} \cdot E\left[\sum_i \left(\frac{\sum_j I(\eta_i^{(1)}, \eta_j^{(0)})}{n_0} - \hat{C}\right)^2\right] \\
=& \frac{1}{n_1^2} E\left[\sum_i \left(\frac{\sum_j I(\eta_i^{(1)}, \eta_j^{(0)})}{n_0}\right)^2\right] - 2\frac{C}{n_1} E\left[\frac{\sum_i \sum_j I(\eta_i^{(1)}, \eta_j^{(0)})}{n_0 n_1}\right] + \frac{n_1 C^2}{n_1^2} \\
=& \frac{1}{n_1^2} E\left[\sum_i \left(\frac{\sum_j I(\eta_i^{(1)}, \eta_j^{(0)})}{n_0}\right)^2\right] - \frac{2C^2}{n_1} + \frac{C^2}{n_1} \\
=& \frac{1}{n_1^2} E\left[\sum_i \left(\frac{\sum_j I(\eta_i^{(1)}, \eta_j^{(0)})}{n_0}\right)^2\right] - \frac{C^2}{n_1}
\end{aligned}$$

Similarly,

$$\frac{E(S_{01})}{n_0} \approx \frac{1}{n_0^2} E\left[\sum_j \left(\frac{\sum_i I(\eta_j^{(0)}, \eta_i^{(1)})}{n_1}\right)^2\right] - \frac{C^2}{n_0} \tag{6}$$

Therefore, equation (5) becomes,

$$
\begin{aligned}
E(\widehat{\mathrm{var}}(\hat{C})) =& \frac{1}{n_1^2}\cdot E\left[\sum_i \left(\frac{\sum_j I(\eta_i^{(1)},\eta_j^{(0)})}{n_0}\right)^2\right] + \frac{1}{n_0^2}\cdot E\left[\sum_j \left(\frac{\sum_i I(\eta_j^{(0)},\eta_i^{(1)})}{n_1}\right)^2\right] - C^2\left(\frac{1}{n_0}+\frac{1}{n_1}\right) &(7)\\
=& \frac{1}{n_1^2}\cdot\left(E\left[\left(\frac{\sum_j I(\eta_1^{(1)},\eta_j^{(0)})}{n_0}\right)^2\right] + \ldots + E\left[\left(\frac{\sum_j I(\eta_{n_1}^{(1)},\eta_j^{(0)})}{n_0}\right)^2\right]\right) + \\
& \frac{1}{n_0^2}\cdot\left(E\left[\left(\frac{\sum_i I(\eta_1^{(0)},\eta_i^{(1)})}{n_1}\right)^2\right] + \ldots + E\left[\left(\frac{\sum_i I(\eta_{n_0}^{(0)},\eta_i^{(1)})}{n_1}\right)^2\right]\right) - C^2\left(\frac{1}{n_0}+\frac{1}{n_1}\right) &(8)\\
=& \frac{1}{n_1^2}\cdot\left(E\left[E_{\eta^{(0)}}\left(I(\eta_1^{(1)},\eta^{(0)})\right)^2\right] + \ldots + E\left[E_{\eta^{(0)}}\left(I(\eta_{n_1}^{(1)},\eta^{(0)})\right)^2\right]\right) + \\
& \frac{1}{n_0^2}\cdot\left(E\left[E_{\eta^{(1)}}\left(I(\eta_1^{(0)},\eta^{(1)})\right)^2\right] + \ldots + E\left[E_{\eta^{(1)}}\left(I(\eta_{n_0}^{(0)},\eta^{(1)})\right)^2\right]\right) - C^2\left(\frac{1}{n_0}+\frac{1}{n_1}\right) &(9)\\
=& \frac{1}{n_1^2}\cdot\left(E\left[P\left(\eta^{(0)}<\eta_1^{(1)}\right)^2\right] + \ldots + E\left[P\left(\eta^{(0)}<\eta_{n_1}^{(1)}\right)^2\right]\right) + \\
& \frac{1}{n_0^2}\cdot\left(E\left[P\left(\eta^{(1)}>\eta_1^{(0)}\right)^2\right] + \ldots + E\left[P\left(\eta^{(1)}>\eta_{n_0}^{(0)}\right)^2\right]\right) - C^2\left(\frac{1}{n_0}+\frac{1}{n_1}\right) &(10)\\
=& \frac{1}{n_1^2}\cdot\left(E_{\eta^{(0)}}\left[K^2(\eta_1^{(1)})\right] + \ldots + E_{\eta^{(0)}}\left[K^2(\eta_{n_1}^{(1)})\right]\right) + \\
& \frac{1}{n_0^2}\cdot\left(E_{\eta^{(1)}}\left[\left((1-G(\eta_1^{(0)})\right)^2\right] + \ldots + E_{\eta^{(1)}}\left[(1-G(\eta_{n_0}^{(0)}))^2\right]\right) - C^2\left(\frac{1}{n_0}+\frac{1}{n_1}\right) &(11)\\
=& \frac{1}{n_1}E_{\eta^{(1)}}\left(K^2(\eta^{(1)}\right) + \frac{1}{n_0}E_{\eta^{(0)}}\left(1-G(\eta^{(0)})\right)^2 - C^2\left(\frac{1}{n_0}+\frac{1}{n_1}\right) &(12)\\
=& \frac{1}{np}E_{\eta^{(1)}}\left(K^2(\eta^{(1)}\right) + \frac{1}{n-np}E_{\eta^{(0)}}\left(\left(1-G(\eta^{(0)}\right)^2\right) - C^2\left(\frac{1}{n-np}+\frac{1}{np}\right)
\end{aligned}
$$

$$
E(\widehat{\mathrm{var}}(\hat{C})) = \frac{1}{n}\times\frac{(1-p)E_{\eta^{(1)}}\left(K^2(\eta^{(1)}\right) + pE_{\eta^{(0)}}\left(1-G(\eta^{(0)})\right)^2 - C^2}{p(1-p)} \tag{13}
$$

where G and K are the cumulative distribution functions of the linear predictor for the cases and controls, respectively. So, $G(\eta^{(0)}) = P(\eta^{(1)} < \eta^{(0)})$ and $K(\eta^{(1)}) = P(\eta^{(0)} < \eta^{(1)})$. Then (13) is the asymptotic variance of the C-statistic.

Given an arbitrary (marginal) distribution F of the linear predictor with density funciton f, the distribution of the linear predictor for cases and controls, respectively, has been given by Gail and Pfeiffer (2005):

$$
G(x) = P(\eta^{(1)} \leq x) = \frac{\int_{-\infty}^{x}\pi(\eta)f(\eta)d\eta}{\int_{-\infty}^{\infty}\pi(\eta)f(\eta)d\eta} \tag{14}
$$

$$
K(x) = P(\eta^{(0)} \leq x) = \frac{\int_{-\infty}^{x}(1-\pi(\eta))f(\eta)d\eta}{\int_{-\infty}^{\infty}(1-\pi(\eta))f(\eta)d\eta}. \tag{15}
$$

These probability distributions can be obtained using numerical integration, after assuming a functional form for the probability density function of η. Having obtained these probability distributions, the expectations in (13) can also be computed using numerical integration.

In practice, risk models most often include a number of continuous and categorical predictors, and, unless this number is very small or there are only binary predictors with extreme prevalences, the distribution of η is likely to be approximately marginally Normal.

Assumption 1: Marginal normality of the linear predictor

$$\eta \sim N(\mu, \sigma^2)$$

In applying equation (13) under the assumption of marginal normality, values for the parameters of μ and σ^2 need to be chosen to match the anticipated values of the outcome prevalence and C-statistic. To avoid the use of simulation in choosing suitable values for μ and σ^2, we obtain in the next subsection the following expressions for μ and σ^2

$$\mu \approx \frac{\sigma_c^2}{2}(2p-1) + log\left(\frac{p}{1-p}\right), \tag{16}$$

and

$$\sigma^2 \approx p^2\sigma_c^2 + (1-p)^2\sigma_c^2. \tag{17}$$

that correspond approximately to the required anticipated values of C and p. We also show that the approximation works very well for a wide range of values of C and p (within 1.5% of the required anticipated values in all scenarios). More information for cross-checking that these values are adequate is given in the Supplementary Material 3.

To obtain a simpler estimator of the variance of $\hat{C}$ that does not depend on patient-level data and involves less computation, we alternatively assume that the linear predictor is Normally distributed conditionalal on Y.

Assumption 2: Conditional Normality of the linear predictor

$$\eta_j^{(0)} \sim N(\mu_0, \sigma_0^2)$$
$$\eta_i^{(1)} \sim N(\mu_1, \sigma_1^2)$$

Under Assumption 1, the assumption of conditional normality for the distribution of the linear predictor, a simple expression in closed form can be conveniently obtained for the variance of the estimated C-statistic, by substituting K and G in (13) by the cumulative probability function of the Normal distribution.

$$E(\widehat{\text{var}}(\hat{C}) = \frac{1}{n} \times \frac{(1-p)E_{\eta^{(1)}}\left(\Phi\left(\frac{\eta^{(1)}-\mu_0}{\sigma_0}\right)^2\right) + pE_{\eta^{(0)}}\left(\left(1-\Phi\left(\frac{\eta^{(0)}-\mu_1}{\sigma_1}\right)\right)^2\right) - C^2}{p(1-p)} \tag{18}$$

where Φ is the standard Normal CDF. $E_{\eta^{(1)}}\left[\Phi\left(\frac{\eta^{(1)}-\mu_0}{\sigma_0}\right)^2\right]$ can be evaluated by

$$E_{\eta^{(1)}}\left[\Phi\left(\frac{\eta^{(1)}-\mu_0}{\sigma_0}\right)^2\right] = \int_{-\infty}^{\infty}\Phi\left(\frac{\eta^{(1)}-\mu_0}{\sigma_0}\right)^2\phi(\eta^{(1)})d\eta^{(1)} = \Phi\left(\frac{\mu_1-\mu_0}{\sqrt{\sigma_1^2+\sigma_0^2}}\right) - 2T\left(\frac{\mu_1-\mu_0}{\sqrt{\sigma_1^2+\sigma_0^2}}, \frac{1}{\sqrt{3}}\right) \tag{19}$$

where T is Owen's T function. Similarly,

$$E_{\eta^{(0)}}\left[\left(1-\Phi\left(\frac{\eta^{(0)}-\mu_1}{\sigma_1}\right)\right)^2\right] = 1-\Phi\left(\frac{\mu_0-\mu_1}{\sqrt{\sigma_1^2+\sigma_0^2}}\right) - 2T\left(\frac{\mu_0-\mu_1}{\sqrt{\sigma_1^2+\sigma_0^2}}, \frac{1}{\sqrt{3}}\right) \tag{20}$$

Substituting equation (19) and (20) into equation (18) we obtain

$$\begin{aligned} E(\widehat{\text{var}}(\hat{C})) = \frac{1}{np}&\left(\Phi\left(\frac{\mu_1-\mu_0}{\sqrt{\sigma_1^2+\sigma_0^2}}\right) - 2T\left(\frac{\mu_1-\mu_0}{\sqrt{\sigma_1^2+\sigma_0^2}}, \frac{1}{\sqrt{3}}\right)\right) \\ &+ \frac{1}{n(1-p)}\left(1-\Phi\left(\frac{\mu_0-\mu_1}{\sqrt{\sigma_1^2+\sigma_0^2}}\right) - 2T\left(\frac{\mu_0-\mu_1}{\sqrt{\sigma_1^2+\sigma_0^2}}, \frac{1}{\sqrt{3}}\right)\right) - \frac{C^2}{n(p-p^2)}. \end{aligned} \tag{21}$$

Under Assumption 2, the $C-$statistic can be approximated by $C = \Phi\left(\frac{\mu_1-\mu_0}{\sqrt{\sigma_1^2+\sigma_0^2}}\right)$ (Zhou (2002)).

Hence, from equation(21) the variance estimator under Assumption 1 is:

$$\begin{aligned} \widehat{\text{var}}_{app}(\hat{C}) &= \frac{1}{np}\left(C-2T\left(\Phi^{-1}(C), \frac{1}{\sqrt{3}}\right)\right) + \frac{1}{n(1-p)}\left(1-C-2T\left(\Phi^{-1}(C), \frac{1}{\sqrt{3}}\right)\right) - \frac{C^2}{n(p-p^2)} \\ \widehat{\text{var}}_{app}(\hat{C}) &= \frac{1}{n} \times \frac{C-2T(\Phi^{-1}(C), \frac{1}{\sqrt{3}})-C^2}{p-p^2}. \end{aligned} \tag{22}$$

As we demonstrate later, conditional normality of the linear predictor also correponds to marginal normality of the linear predictor for values of the C-statistic approximately up to 0.9. As we show in simulations in the main paper, our derived formula performs very well when the linear predictor is normally distributed, which is a realistic assumption that is likely to hold in practice.

Proof of the formulae for the values of μ and σ^2 under marginal normality

Assuming that the distribution of the linear predictor is Normal with parameters μ and σ^2, in applying (13) values for these parameters can be chosen by simulation to correspond to the anticipated p and C.

However, we note that when the conditional distribution of the linear predictor (given the outcome) is Normal with common variance in the cases and controls groups, then the marginal distribution of the linear predictor

is also approximately Normal when C is not too large (<0.9). This can be seen in the Figure (1) below for p=0.1 and values of C between 0.64-0.98. Hence, for values of $C < 0.9$, the marginal linear predictor can be reasonably approximated as the mixture of two conditionally Normal (on the outcome) distributions with common variance.

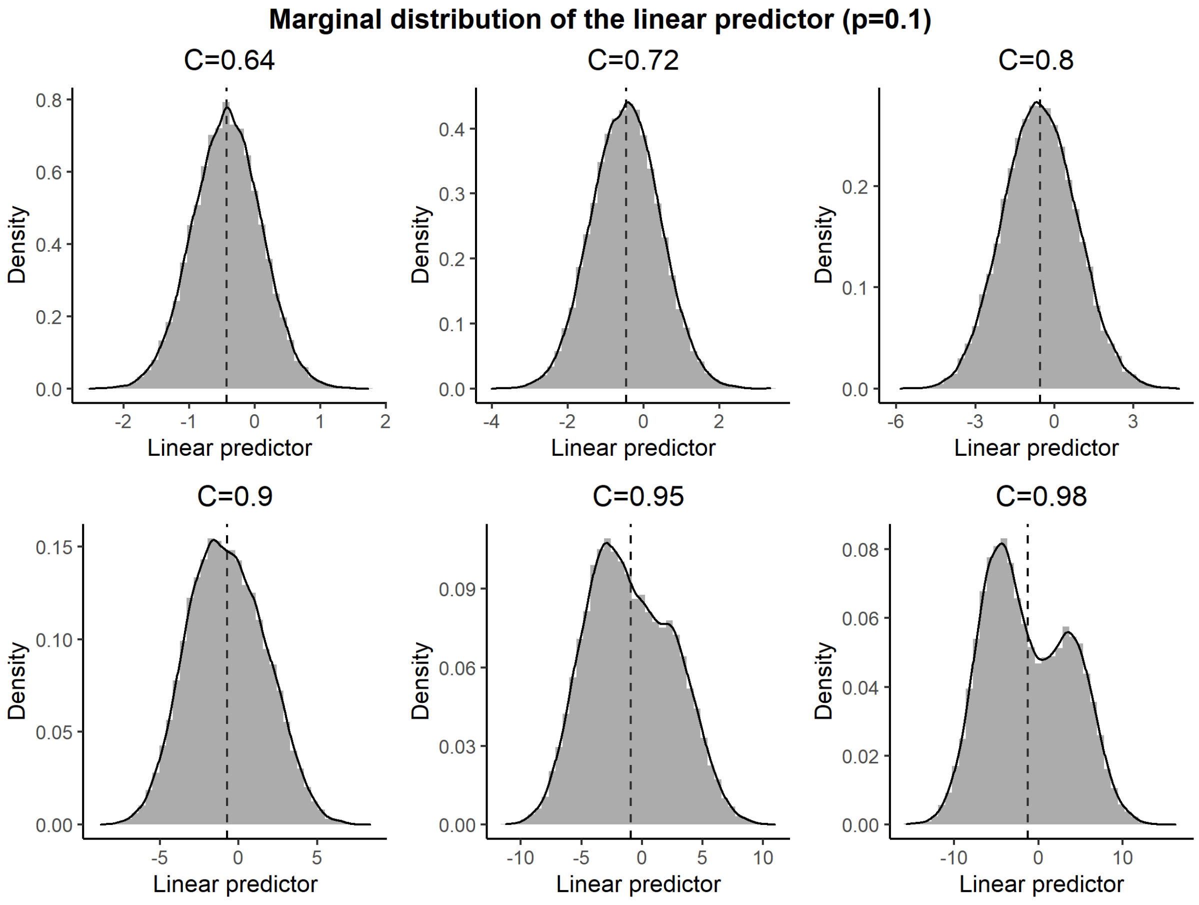

Figure 1: Marginal distribution of the linear predictor when the conditional distribution of the linear predictor given the outcome is Normal with equal variance in the cases and controls groups.

Letting $\eta^{(1)} \sim N(\mu_1, \sigma_c^2)$ and $\eta^{(0)} \sim N(\mu_0, \sigma_c^2)$ denote the conditional distribution of the linear predictor in the cases and control groups, respectively, the marginal distribution of the linear predictor, η, can be approximated by

$$\eta \approx p\,\eta^{(1)} + (1-p)\eta^{(0)}. \tag{23}$$

We first note that $C \approx \Phi\left(\dfrac{\mu_1 - \mu_0}{\sigma\sqrt{2}}\right)$. Using the relationship between the parameters in a logistic regression

model for the calibration parameters in the model

$$\text{logit}(\pi_i) = \text{logit}(P(Y_i = 1|\eta_i) = \alpha + \beta\eta_i, \tag{24}$$

and the corresponding LDA model (e.g. Efron (1975)), the parameters in model (24) can be expressed as

$$\alpha = -log(n_1/n_0) + \frac{\mu_1^2 - \mu_0^2}{2\sigma^2} \text{ and } \beta = \frac{\mu_1 - \mu_0}{\sigma^2} \tag{25}$$

Assuming a well-calibrated model with $\alpha = 0$ and $\beta = 1$ in model (24), then

$$\mu_1 = \frac{\sigma_c^2}{2} + log\left(\frac{n_1}{n_0}\right), \tag{26}$$

$$\mu_0 = \mu_1 - \sigma_c^2 \text{ and} \tag{27}$$

$$\sigma_c = \sqrt{2}\Phi^{-1}(C) \tag{28}$$

Using (23) we can approximate the mean and the variance of the marginally normally distributed linear predictor, given p and C:

$$\begin{aligned} \mu =& E(\eta) \approx pE(\eta^{(1)}) + (1-p)E(\eta^{(0)}) \\ =& p\left(\frac{\sigma_c^2}{2} + log\left(\frac{n_1}{n_0}\right)\right) + (1-p)\left(\frac{\sigma_c^2}{2} + log\left(\frac{n_1}{n_0}\right) - \sigma_c^2\right) \Rightarrow \\ \mu \approx& \frac{\sigma_c^2}{2}(2p-1) + log\left(\frac{p}{1-p}\right), \end{aligned} \tag{29}$$

and

$$\begin{aligned} \sigma^2 =& var(\eta) \approx p^2 var(\eta^{(1)}) + (1-p)^2 var(\eta^{(0)}) \Rightarrow \\ \sigma^2 \approx& p^2\sigma_c^2 + (1-p)^2\sigma_c^2. \end{aligned} \tag{30}$$

In practice, the selected values of μ and σ^2, should correspond to the anticipated C and p. For given values of μ and σ^2, the actual p and C can be computed using simulation and the following steps:

1. Set the anticipated p and C. These are the input values in the step below.
2. Compute μ and σ^2 using (29) and (30) or any other way
3. Generate M samples from the distribution of the linear predictor $\eta \sim N(\mu, \sigma^2)$ where M is large (e.g. $M = 1 \times 10^6$)
4. Generate M binary responses, $Y \mid \eta \sim Bernoulli(logit^{(-1)}(\eta))$,
5. Using the the M realisations of η and Y calculate the actual, true prevalence and C-statistic that correspond to the chosen values of μ and σ^2, and compare with the desired, anticipated ones.

To check the quality of the chosen values for μ and σ^2 using (29) and (30) we apply steps (1)-(5) above for a range of values for the anticpiated, input values of C and p. The results presented in Table (1) show that the actual p and C for the chosen values of μ and σ^2 are very close to the anticipated, input C-statistic and prevalence. Hence, for the purposes of sample size calculations, (29) and (30) can be reliably used to detect the values of μ and σ^2 that correspond to the desired C and p, avoiding the need for trial and error. Specifically, the agreement is remarkably good for values of C up 0.8 (within 0.5 % of the true value), while for a $C >= 0.85$, the disagreement increases slightly (up to 1.5 % of the true value when $C = 0.9$). Should it be required, this minor disagreement for high values of C can be resolved by slightly inflating σ_c in (28) by a factor f_c. Inflating by a factor of 1.02-1.03 when C=0.85 and 1.03-1.05 when C=0.9, will provide actual values that are closer to the required anticipated values. More details, and the code to perform these checks are given in Supplementary material 3.

Anticipated p	Anticipated C	μ	σ	Actual p	Actual c
0.05	0.64	-3.06	0.51	0.050	0.640
0.05	0.72	-3.25	0.84	0.050	0.719
0.05	0.80	-3.58	1.23	0.050	0.797
0.05	0.85	-3.91	1.54	0.049	0.844
0.05	0.90	-4.42	1.95	0.048	0.889
0.10	0.64	-2.30	0.51	0.100	0.640
0.10	0.72	-2.47	0.85	0.100	0.719
0.10	0.80	-2.76	1.26	0.100	0.796
0.10	0.85	-3.06	1.60	0.099	0.843
0.10	0.90	-3.51	2.06	0.098	0.888
0.30	0.64	-0.90	0.52	0.300	0.640
0.30	0.72	-0.98	0.88	0.300	0.720
0.30	0.80	-1.13	1.36	0.301	0.797
0.30	0.85	-1.28	1.77	0.301	0.845
0.30	0.90	-1.50	2.36	0.303	0.891
0.40	0.64	-0.43	0.52	0.400	0.639
0.40	0.72	-0.47	0.89	0.400	0.719
0.40	0.80	-0.55	1.38	0.401	0.798
0.40	0.85	-0.62	1.80	0.402	0.845
0.40	0.90	-0.73	2.42	0.403	0.892
0.50	0.64	0.00	0.52	0.500	0.640
0.50	0.72	0.00	0.89	0.500	0.720
0.50	0.80	0.00	1.38	0.500	0.799
0.50	0.85	0.00	1.82	0.500	0.846
0.50	0.90	0.00	2.45	0.499	0.892

Table 1: Calculation of actual values of p and C when μ and σ^2 were chosen using (29) and (30). The actual p and C, for the purposes of sample size calculations are sufficiently close to the required anticipated values.

2 Proof of the closed-form formula for the variance of the estimated calibration in the large

The calibration in the large is the intercept term in the following logistic regression model:

$$\text{logit}(\pi_i) = \text{logit}(P(Y_i = 1|\eta_i) = \alpha_{CL} + \eta_i, \tag{31}$$

which is equivalent to model (36), with the coefficient of the calibration slope set to 1 (i.e. the estimated linear predictor is included as an offset term).

The variance of the estimated calibration in the large can be obtained as the asymptotic approximation to the inverse of Fisher's information in model (31)

$$\widehat{\text{var}}_{MC}(\hat{\alpha}_{CL}) = \frac{1}{nE(W)}, \tag{32}$$

where $W = \pi(1-\pi)$, $\pi = (1 + exp(-\eta))^{-1}$, and η is assumed to follow a distribution F with parameters θ.

An estimator of $\hat{\alpha}_{CL}$ is

$$\widehat{\text{var}}(\hat{\alpha}_{CL}) = \frac{1}{\sum_{i=1}^{n} w_i} \tag{33}$$

where $w_i = \pi_i(1-\pi_i)$ and $\pi_i = (1 + exp(-\eta_i))^{-1}$ and n is the sample size.

Assuming that the distribution of η is Normal with mean μ and variance σ^2 (Assumption 1)we use Taylor approximations to obtain a closed for expression for $E(W)$, in terms of μ and σ^2 only.

The Taylor expansion of $w = g(\eta) = p(1-p)$ around $\eta = \mu$ is

$$g(\eta) \approx g(\mu) + g'(\mu)(\eta-\mu) + \frac{1}{2}g''(\mu)(\eta-\mu)^2 + \frac{1}{6}g'''(\mu)(\eta-\mu)^3 + ...$$

Taking the expectation of the expression above, the odd central moments of η are zero, hence the expectation of the Taylor expansion up to order 3 is:

$$E(W) = E(g(\eta)) \approx g(\mu) + \frac{1}{2}g''(\mu)E(\eta-\mu)^2 = g(\mu) + g''(\mu)\sigma^2. \tag{34}$$

We note that equation (34) is also true for any distribution for the linear predictor with mean μ and variance σ^2, assuming that the terms above order 2 in the Taylor approximation are zero.

Applying the chain rule for $\frac{dg}{d\eta}$ where $\frac{\partial\pi}{\partial\eta} = \frac{\partial\left(\frac{\exp(\eta)}{1+\exp(\eta)}\right)}{\partial\eta} = \frac{\exp(\eta)}{1+\exp(\eta)}\frac{1}{1+\exp(\eta)} = \pi(1-\pi) = \pi - \pi^2$ we obtain

$$\frac{dg}{d\eta} = \frac{\partial g}{\partial \pi}\frac{\partial \pi}{\partial \eta} = (1-2\pi)(\pi - \pi^2) = -3\pi^2 + 2\pi^3 + \pi, \text{ and}$$

$$\frac{d^2 g}{d\eta^2} = \frac{\partial^2 g}{\partial \pi^2}\frac{\partial \pi}{\partial \eta} = (-6\pi + 6\pi^2 + 1)(\pi - \pi^2)$$

Hence,

$$E(W) \approx \tilde{\pi}(1-\tilde{\pi})\left((1 + \frac{1}{2}(1 - 6\tilde{\pi} + 6\tilde{\pi}^2)\sigma^2\right) \tag{35}$$

where $\tilde{\pi} = (1 + exp(\mu))^{-1}$.

Substituting (35) in (32) we obtain an expression for the variance of the estimated calibration in the large that only depends on the sample size, μ and σ^2.

3 Proof of the closed-form formula for the variance of the estimated calibration slope

The calibration slope is the coefficient of the linear predictor in the following logistic regression model:

$$\text{logit}(P(Y_i = 1|\eta_i) = \alpha + \beta_{cs}\eta_i. \tag{36}$$

We aim to obtain an estimator for variance of the estimated calibration slope that is free from patient-level information. To achieve this we start by assuming that the distribution of the linear predictor is conditionally Normal given the binary outcome and that the corresponding variances are equal.

Assumption 3:

$$\eta_i^{(1)} \sim N(\mu_1, \sigma^2)$$
$$\eta_j^{(0)} \sim N(\mu_0, \sigma^2)$$

where $\eta_i^{(1)}$ and$\eta_j^{(0)}$ are defined as above.

Assumption 3 corresponds to the data-generating mechanism from a Linear Discriminant Analysis (LDA) model. We also note that Assumption 3 is very similar to Assumption 2, only additionally requiring that $\sigma_0^2 = \sigma_1^2$. Using the relationship the parameters of an LDA model and a logistic regression model (e.g. Efron (1975)), calibration slope can be estimated by

$$\hat{\beta}_{cs}^{LDA} = \frac{\bar{\eta}_1 - \bar{\eta}_0}{\hat{\sigma}^2} \tag{37}$$

where $\bar{\eta}_0 = \frac{1}{n_0}\sum_i^{n_0} \eta_i^{(0)}$ and $\bar{\eta}_1 = \frac{1}{n_1}\sum_j^{n_1} \eta_j^{(1)}$.
The variance of the estimated calibration slope can be estimated from (37)

$$\widehat{\text{var}}(\hat{\beta}_{cs}^{LDA}) = \text{var}\left(\frac{\bar{\eta}_1 - \bar{\eta}_0}{\hat{\sigma}^2}\right) \tag{38}$$

$$\approx \frac{(\bar{\eta}_1 - \bar{\eta}_0)^2}{(\sigma^2)^2}\left[\frac{\text{var}(\bar{\eta}_1 - \bar{\eta}_0)}{(\bar{\eta}_1 - \bar{\eta}_0)^2} + \frac{\text{var}(\hat{\sigma}^2)}{(\sigma^2)^2}\right] \tag{39}$$

$$\approx \frac{(\bar{\eta}_1 - \bar{\eta}_0)^2}{\sigma^4}\left[\frac{\sigma^2(1/n_1 + 1/n_0)}{(\bar{\eta}_1 - \bar{\eta}_0)^2} + \frac{2\sigma^4}{(n-2)\sigma^4}\right] \tag{40}$$

We obtained (39) from (38) using the following relationship for the variance of the quotient of two random variables A and B (obtained using the first order Taylor expansion): $\text{var}\left(\frac{A}{B}\right) \approx \frac{\mu_A^2}{\mu_B^2}\left[\frac{\sigma_A^2}{\mu_B^2} - 2\frac{\text{Cov}(A,B)}{\mu_A\mu_B} + \frac{\sigma_B^2}{\mu_B^2}\right]$. Subsequently, using that $\text{var}(\bar{\eta}_1 - \bar{\eta}_0) = \sigma^2(1/n_1 + 1/n_0)$ and $\text{var}(\sigma^2) = 2\sigma^4/(n-2)$ we obtained (40) from (39). Finally, since $\hat{C} = \Phi\left(\frac{\mu_1 - \mu_0}{\sqrt{2\sigma^2}}\right)$ and $\hat{\beta}_{cs} = \frac{\bar{\eta}_1 - \bar{\eta}_0}{\sigma^2}$, it follows that $\beta_{cs} = \frac{\sqrt{2}\Phi^{-1}(C)}{\sigma}$ and

$$\sigma = \frac{\sqrt{2}\Phi^{-1}(C)}{\beta_{cs}}. \tag{41}$$

Substituting (41) into (40) we obtain

$$\widehat{\text{var}}(\hat{\beta}_{cs}^{LDA}) \approx \frac{1}{n} \times \frac{\beta_{cs}^2}{2p(1-p)\Phi^{-1}(C)^2} + \frac{2\beta_{cs}^2}{n-2}. \tag{42}$$

This formula for calculating the variance of $\hat{\beta}_{cs}^{logis}$ is valid as long as the variance of $\hat{\beta}_{cs}^{LDA}$ is the same as the variance of the estimated calibration slope, $\hat{\beta}_{cs}^{logis}$, obtained from the fit of model (36). As logistic regression models $P(Y|\eta)$ while LDA models $P(\eta|Y)$ and $P(Y)$, hence using more information than logistic regression, $\widehat{\text{var}}(\hat{\beta}_{cs}^{LDA})$ will, at least asymptotically, be smaller than $\widehat{\text{var}}(\hat{\beta}_{cs}^{logis})$. As noted by Efron(1975) the efficiency of LDA is higher than logistic regression for higher values of C, while the efficiency of the two methods will be similar for values of C up to 0.8-0.85 and prevalence close to 0.5. This is confirmed by a simulation in section 5 to compare the efficiency of the two methods when data are generated under model Assumption 3 (DGM1 of Section 5) and are presented in Figure (2) below. Therefore our variance formula above is expected to work well for this range of values. For very high values of the C-statistic, the variance of the estimated calibration slope obtained from fitting model (36)will tend to be underestimated by equation (42).

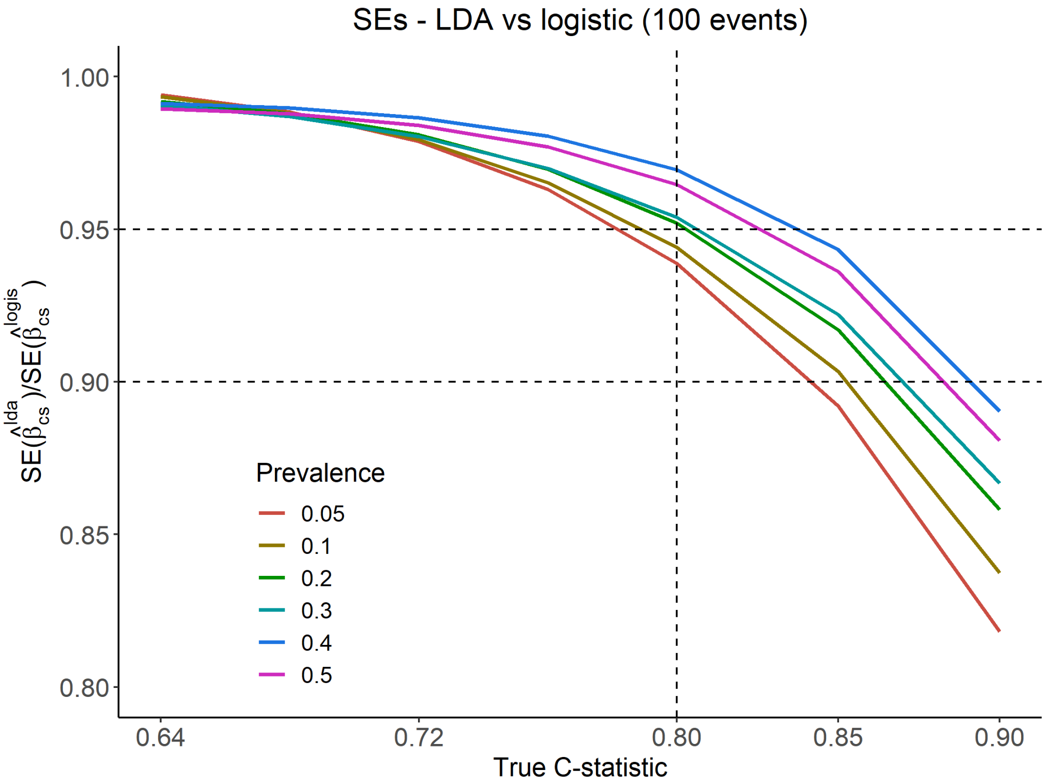

Figure 2: Standard errors for the estimated calibration slope from LDA and logistic regression.
