# Supplementary material for: Estimation of required sample size for external validation of risk models for binary outcomes
Source: Stat Methods Med Res. 2021 Apr 21;30(10):2187–206. doi: 10.1177/09622802211007522 (PMC8529102; doi:10.1177/09622802211007522)

Supplementary material 2 – Additional Simulation Results

1. Simulation of data for DGMs 1-4 (Section 5 of the main paper)

DGM 1: The linear predictor is conditionally Normally distributed given Y with the corresponding variances being equal. Data are generated from a LDA model under Assumption 2. In this simulation Y is fixed, i.e. we fix the number of cases, n_1, and the number of controls, n_0, rather than drawing Y randomly. To achieve this, we first note that $C = \Phi\left(\frac{\mu_1-\mu_0}{\sigma\sqrt{2}}\right)$. Using the relationship between the parameters in a logistic regression model and the corresponding LDA model[22], the parameters in equation (10) of the main paper can be expressed as

$$\alpha = -\log\left(\frac{n_1}{n_0}\right) + \frac{\mu_1^2 - \mu_0^2}{2\sigma^2} \quad \text{and} \quad \beta_{CS} = \frac{\mu_1 - \mu_0}{\sigma^2}.$$

So, $C = \Phi\left(\frac{\sigma\,\beta_{CS}}{\sqrt{2}}\right)$ and solving for σ,

$$\sigma = \frac{\sqrt{2}\,\Phi^{-1}(C)}{\beta_{CS}} \quad . \qquad (*)$$

Assuming that $\alpha = 0$ and $\beta_{CS} = 1$ for a well calibrated model:

$$\mu_1 = \frac{\sigma^2}{2} + \log\frac{n_1}{n_0}$$

$$\mu_0 = \mu_1 - \sigma^2$$

Values of σ^2, μ_0 and μ_1 are chosen to achieve the required p and C. We note that when the distribution of the linear predictor is conditionally Normal given the outcome and the corresponding variances equal, the marginal distribution of the linear predictor is also approximately Normal when $\mu_1 - \mu_2$ is small relative to σ, corresponding to values of C up to 0.9 (see Figure S2).

DGM 2: The linear predictor is conditionally Normally distributed given the outcome

$$\eta | Y \sim N(\mu_Y, \sigma_Y^2) \text{ and } Y = 0, 1$$

and $\sigma_0 \neq \sigma_1$. To generate a linear predictor with unequal variances for cases and controls we use $\sigma_0 = 1.33 \times \sigma^*$ and $\sigma_1 = 0.67 \times \sigma^*$, where $\sigma^* = \sqrt{2}\,\Phi^{-1}(C)$ as in equation (*) with $\beta_{CS} = 1$. We then use $\mu_1 = \frac{\sigma^2}{2} + \log\left(\frac{n_1}{n_0}\right)$ and $\mu_0 = \mu_1 - \sigma^2 + k$. Contrary to DGM 1, Y is not fixed; the binary outcomes are

generated from the following logistic regression: $\text{logit}\big(P(Y = 1)\big) = \eta$. Values of $\sigma_1, \sigma_2,\ \mu_0, \mu_1$ and k are chosen to achieve the required p and C.

DGM 3: The marginal distribution of the linear predictor is Normal: $\eta \sim N(\mu, \sigma^2)$. The binary outcomes are generated from the following logistic regression model: $\text{logit}\big(P(Y = 1)\big) = \eta$. Values for μ and σ^2 were chosen to generate data with the required p and C. More precisely, for values of anticipated C up to 0.8, μ and σ^2 were calculated directly using (7) and (8) in Section 3.1. For higher values of C, the values of μ and σ^2 provided by (7) and (8) were adjusted to give values of C and p closer to the required anticipated ones. It is noted that the resulting conditional distribution of the linear predictor given the outcome is also approximately Normal, and the corresponding variances are very similar, for values of C up to 0.8 (see Figure S3).

DGM 4: The linear predictor with a skewed marginal distribution is generated as the linear combination of 4 independent binary variables with prevalences 0.1, 0.2, 0.3 and 0.5, respectively: $\eta = \beta_0 + \beta_1 X_1 + \beta_2 X_2 + \beta_3 X_3 + \beta_4 X_4$. The binary outcomes are then generated from the model $\text{logit}\big(P(Y = 1 \mid X_1, X_2, X_3, X_4)\big) = \eta$. Values for the regression coefficients $\beta_0, \beta_1, \beta_2, \beta_3, \beta_4$ were varied to achieve the required p and C. The conditional distribution of the linear predictor given the outcome is also highly skewed and the variances for cases and controls are different (see Figure S4).

2. Simulation setting for Survival data (Section 6 of the main paper)

The survival function of the proportion hazards is given by

$S(t|x) = \exp\,[-H_0(t) \times \exp(\beta' x)]$

The linear predictor was generated from a Normal distribution, varying the parameters to achieve the desired values of C-index and prevalence.

Let baseline hazard $h_0(t) = \lambda \rho t^{\rho - 1}$ from *Weibull*(λ,ρ) with shape ρ = 0.0009 and scale λ=1. Then we have $H_0(t) = \lambda t^{\rho}$ which is invertible, so that $H_0^{-1}(t) = (\frac{t}{\lambda})^{\frac{1}{\rho}}$. The survival time T can be generated by,

$$t = (-\frac{\log\,(v)}{\lambda \exp\,(x'\beta)})^{\frac{1}{\rho}}$$

Where v is a random variable with V~ Uni[0,1]. Censoring time C was generated from an exponential distribution.

3. Tables and Figures

Table S1. DGM1. % Bias of $SE_{app}(\hat{C})$ and $SE_{app}(\hat{\beta}_{CS})$ calculated over 10,000 simulations for true prevalence values 5%, 10% and 30% and true C-statistic of 0.64, 0.72, 0.8, 0.85 and 0.9.

			C-statistic			Calibration slope		
p	C	n_{events}	SE_{true}	SE_{app}	% Bias (SE_{app})	SE_{true}	SE_{app}	% Bias (SE_{app})
0.05	0.64	50	0.0401	0.0398	-1	0.2927	0.2897	-1
		100	0.0284	0.0281	-1	0.2075	0.2048	-1
		200	0.0201	0.0199	-1	0.1474	0.1448	-2
		400	0.0141	0.0141	-1	0.1031	0.1024	-1
0.05	0.72	50	0.0366	0.0365	0	0.1863	0.1816	-2
		100	0.0260	0.0258	-1	0.1319	0.1284	-3
		200	0.0185	0.0183	-1	0.0937	0.0908	-3
		400	0.0130	0.0129	-1	0.0655	0.0642	-2
0.05	0.8	50	0.0316	0.0315	0	0.1385	0.1299	-6
		100	0.0224	0.0223	-1	0.098	0.0918	-6
		200	0.0159	0.0158	-1	0.0696	0.0649	-7
		400	0.0112	0.0111	-1	0.0485	0.0459	-5
0.05	0.85	50	0.0273	0.0272	0	0.1218	0.1086	-11
		100	0.0194	0.0192	-1	0.0861	0.0768	-11
		200	0.0138	0.0136	-1	0.0611	0.0543	-11
		400	0.0097	0.0096	-1	0.0425	0.0384	-10
0.05	0.9	50	0.0218	0.0217	0	0.1126	0.0917	-19
		100	0.0155	0.0154	-1	0.0793	0.0649	-18
		200	0.011	0.0109	-1	0.0560	0.0459	-18
		400	0.0077	0.0077	0	0.0388	0.0324	-16
0.1	0.64	50	0.0416	0.0409	-2	0.3091	0.3008	-3
		100	0.0290	0.0289	0	0.2150	0.2127	-1
		200	0.0206	0.0204	-1	0.1526	0.1504	-1
		400	0.0145	0.0144	0	0.1080	0.1063	-2
0.1	0.72	50	0.0377	0.0375	0	0.2007	0.1916	-5
		100	0.0266	0.0265	0	0.1391	0.1355	-3
		200	0.0189	0.0188	-1	0.0984	0.0958	-3
		400	0.0133	0.0133	0	0.0698	0.0677	-3
0.1	0.8	50	0.0325	0.0324	-1	0.1533	0.1404	-8
		100	0.0230	0.0229	0	0.1059	0.0992	-6
		200	0.0162	0.0162	0	0.0747	0.0702	-6

		400	0.0115	0.0114	0	0.0530	0.0496	-6
0.1	0.85	50	0.0281	0.028	-1	0.1374	0.1198	-13
		100	0.0199	0.0198	-1	0.0947	0.0847	-11
		200	0.014	0.014	0	0.0667	0.0599	-10
		400	0.0099	0.0099	-1	0.0472	0.0423	-10
0.1	0.9	50	0.0225	0.0223	-1	0.1294	0.1038	-20
		100	0.0159	0.0158	-1	0.0889	0.0734	-17
		200	0.0112	0.0112	-0	0.0623	0.0519	-17
		400	0.0079	0.0079	-1	0.0440	0.0367	-17
0.3	0.64	50	0.0481	0.0463	-4	0.3685	0.3512	-5
		100	0.0327	0.0328	0	0.2522	0.2482	-2
		200	0.0234	0.0232	-1	0.1790	0.1755	-2
		400	0.0162	0.0164	1	0.1236	0.1241	0
0.3	0.72	50	0.0431	0.0426	-1	0.2478	0.2328	-6
		100	0.0300	0.0301	0	0.1690	0.1645	-3
		200	0.0215	0.0213	-1	0.1195	0.1163	-3
		400	0.0149	0.0150	1	0.0826	0.0822	0
0.3	0.8	50	0.0372	0.0367	-1	0.1978	0.1798	-9
		100	0.0259	0.0260	0	0.1340	0.127	-5
		200	0.0185	0.0184	-1	0.0942	0.0897	-5
		400	0.0129	0.0130	1	0.0652	0.0634	-3
0.3	0.85	50	0.0321	0.0317	-1	0.1829	0.1595	-13
		100	0.0224	0.0224	0	0.1229	0.1126	-8
		200	0.016	0.0159	-1	0.0859	0.0796	-7
		400	0.0112	0.0112	0	0.0596	0.0563	-6
0.3	0.9	50	0.0257	0.0253	-1	0.1785	0.1444	-19
		100	0.0179	0.0179	0	0.1181	0.1019	-14
		200	0.0127	0.0126	-1	0.0820	0.072	-12
		400	0.0089	0.0089	0	0.0570	0.0509	-11

Table S2. DGM1. Number of events required to attain a specified standard error for $\hat{C}$ and $\hat{\beta}_{cs}$. % Bias of the estimated sample size (number of events) is calculated over 10,000 simulations for true prevalence values of 5%, 10% and 30% and true C-statistic of 0.64, 0.72, 0.8, 0.85 and 0.9.

		C-Statistic				Calibration Slope			
p	C	SE_{req}	$\hat{n}^{(e)}{}_{\text{req}}$	$n^{(e)}_{\text{req,app}}$	% Bias	SE_{req}	$\hat{n}^{(e)}{}_{\text{req}}$	$n^{(e)}_{\text{req,app}}$	% Bias
0.05	0.64	0.0125	513	507	-1	0.10	420	420	0
0.05	0.72	0.0125	432	427	-1	0.10	169	165	-2
0.05	0.80	0.0125	322	318	-1	0.10	94	85	-10
0.05	0.85	0.0125	231	238	3	0.10	73	59	-19
0.05	0.90	0.0125	153	151	-1	0.10	63	43	-32
0.10	0.64	0.0125	541	535	-1	0.10	453	453	0
0.10	0.72	0.0125	456	451	-1	0.10	188	184	-2
0.10	0.80	0.0125	340	336	-1	0.10	117	99	-15
0.10	0.85	0.0125	249	251	1	0.10	89	72	-19
0.10	0.90	0.0125	156	160	3	0.10	79	54	-32
0.30	0.64	0.0125	695	687	-1	0.10	616	616	0
0.30	0.72	0.0125	563	580	3	0.10	277	271	-2
0.30	0.80	0.0125	437	432	-1	0.10	178	161	-10
0.30	0.85	0.0125	326	322	-1	0.10	148	127	-14
0.30	0.90	0.0125	208	205	-1	0.10	138	104	-25
0.05	0.64	0.025	129	127	-2	0.15	187	187	0
0.05	0.72	0.025	107	107	0	0.15	76	74	-3
0.05	0.80	0.025	78	80	3	0.15	42	38	-10
0.05	0.85	0.025	59	60	2	0.15	34	27	-21
0.05	0.90	0.025	38	38	0	0.15	30	19	-37
0.10	0.64	0.025	136	134	-1	0.15	202	202	0
0.10	0.72	0.025	112	113	1	0.15	84	82	-2
0.10	0.80	0.025	84	84	0	0.15	51	44	-14
0.10	0.85	0.025	63	63	0	0.15	42	32	-24
0.10	0.90	0.025	40	40	0	0.15	37	24	-35
0.30	0.64	0.025	174	172	-1	0.15	274	274	0
0.30	0.72	0.025	147	145	-1	0.15	124	121	-2
0.30	0.80	0.025	108	108	0	0.15	80	72	-10
0.30	0.85	0.025	80	81	1	0.15	70	57	-19
0.30	0.90	0.025	52	52	0	0.15	67	46	-31

Table S3. DGM1. Type I error and power to detect a statistically significant difference, d, from a target value of the C-statistic calculated over 10,000 simulations for true prevalence values 5%, 10% and 30% and true C-statistic of 0.64, 0.72 and 0.8 when $\hat{n}_{req,app}$ is chosen to give 90% power. $\hat{n}^{(e)}{}_{req,app}$ denotes the required number of events.

d	p	C_0	C_1	$\hat{n}^{(e)}{}_{\text{req,app}}$	Type I error	Power
		0.64	0.67	736	0.05	0.90
0.03	0.05	0.72	0.75	610	0.06	0.91
		0.80	0.83	441	0.06	0.90
		0.64	0.67	777	0.05	0.90
0.03	0.10	0.72	0.75	644	0.06	0.90
		0.80	0.83	466	0.06	0.91
		0.64	0.67	999	0.05	0.90
0.03	0.30	0.72	0.75	828	0.05	0.90
		0.80	0.83	599	0.06	0.91
		0.64	0.69	261	0.05	0.90
0.05	0.05	0.72	0.77	213	0.06	0.91
		0.80	0.85	151	0.06	0.91
		0.64	0.69	275	0.06	0.90
0.05	0.10	0.72	0.77	225	0.06	0.91
		0.80	0.85	159	0.07	0.92
		0.64	0.69	354	0.05	0.90
0.05	0.30	0.72	0.77	290	0.06	0.91
		0.80	0.85	205	0.06	0.92

Table S4. DGM 2. % Bias of $SE_{app}(\hat{C})$ and $SE_{app}(\hat{\beta}_{CS})$ calculated over 10,000 simulations for true prevalence values 5%, 10% and 30% and true C-statistic of 0.64, 0.72, 0.8, 0.85 and 0.9.

			C-statistic			Calibration Slope		
p	C	n_{events}	$\mathrm{SE_{true}}$	$\mathrm{SE_{app}}$	% Bias	$\mathrm{SE_{true}}$	$\mathrm{SE_{app}}$	% Bias
0.05	0.64	50	0.0411	0.0398	-3	0.2994	0.2897	-3
		100	0.0277	0.0281	1	0.2035	0.2048	1
		200	0.0199	0.0199	0	0.1465	0.1448	-1
		400	0.0142	0.0141	-1	0.1037	0.1024	-1
0.05	0.72	50	0.0372	0.0365	-2	0.1906	0.1816	-5
		100	0.0255	0.0258	1	0.1294	0.1284	-1
		200	0.0183	0.0183	0	0.0932	0.0908	-3
		400	0.0130	0.0129	-1	0.0658	0.0642	-2
0.05	0.8	50	0.0321	0.0315	-2	0.1415	0.1299	-8
		100	0.0220	0.0223	1	0.0965	0.0918	-5
		200	0.0158	0.0158	0	0.0692	0.0649	-6
		400	0.0112	0.0111	-1	0.0487	0.0459	-6
0.05	0.85	50	0.0277	0.0272	-2	0.1244	0.1086	-13
		100	0.019	0.0192	1	0.0851	0.0768	-10
		200	0.0137	0.0136	-1	0.0607	0.0543	-11
		400	0.0097	0.0096	0	0.0426	0.0384	-10
0.05	0.9	50	0.022	0.0217	-1	0.1149	0.0917	-20
		100	0.0152	0.0154	1	0.0785	0.0649	-17
		200	0.0109	0.0109	-1	0.0558	0.0459	-18
		400	0.0077	0.0077	0	0.0389	0.0324	-17
0.1	0.64	50	0.0416	0.0409	-2	0.3038	0.3008	-1
		100	0.0291	0.0289	-1	0.2184	0.2127	-3
		200	0.0201	0.0204	2	0.1486	0.1504	1
		400	0.0141	0.0144	2	0.1059	0.1063	0
0.1	0.72	50	0.0375	0.0375	0	0.1972	0.1916	-3
		100	0.0267	0.0265	-1	0.1413	0.1355	-4
		200	0.0184	0.0188	2	0.0959	0.0958	0
		400	0.0130	0.0133	2	0.0686	0.0677	-1
0.1	0.8	50	0.0325	0.0324	0	0.1511	0.1404	-7
		100	0.0230	0.0229	-1	0.1075	0.0992	-8
		200	0.0158	0.0162	2	0.0732	0.0702	-4
		400	0.0112	0.0114	2	0.0521	0.0496	-5
0.1	0.85	50	0.0282	0.028	-1	0.136	0.1198	-12
		100	0.0199	0.0198	0	0.0959	0.0847	-12
		200	0.0137	0.014	2	0.0656	0.0599	-9
		400	0.0097	0.0099	2	0.0464	0.0423	-9
0.1	0.9	50	0.0225	0.0223	-1	0.1287	0.1038	-19
		100	0.0158	0.0158	0	0.0895	0.0734	-18
		200	0.011	0.0112	2	0.0618	0.0519	-16
		400	0.0078	0.0079	1	0.0433	0.0367	-15

0.3	0.64	50	0.0468	0.0463	-1	0.3622	0.3512	-3
		100	0.0323	0.0328	1	0.2478	0.2482	0
		200	0.0233	0.0232	0	0.1775	0.1755	-1
		400	0.0163	0.0164	0	0.1244	0.1241	0
0.3	0.72	50	0.0423	0.0426	1	0.2438	0.2328	-5
		100	0.0296	0.0301	1	0.1668	0.1645	-1
		200	0.0214	0.0213	0	0.1189	0.1163	-2
		400	0.0149	0.0150	1	0.0829	0.0822	-1
0.3	0.8	50	0.0363	0.0367	1	0.1952	0.1798	-8
		100	0.0256	0.0260	1	0.1328	0.127	-4
		200	0.0185	0.0184	-1	0.0941	0.0897	-5
		400	0.0128	0.0130	1	0.0652	0.0634	-3
0.3	0.85	50	0.0313	0.0317	1	0.1807	0.1595	-12
		100	0.0222	0.0224	1	0.1222	0.1126	-8
		200	0.0159	0.0159	0	0.086	0.0796	-7
		400	0.0111	0.0112	1	0.0594	0.0563	-5
0.3	0.9	50	0.025	0.0253	1	0.1765	0.1444	-18
		100	0.0178	0.0179	1	0.1179	0.1019	-14
		200	0.0127	0.0126	0	0.0822	0.072	-12
		400	0.0088	0.0089	1	0.0567	0.0509	-10

Table S5. DGM3. % Bias of the standard errors for $\hat{C}$, $\hat{\beta}_{cs}$ and $\hat{\alpha}_{CL}$ calculated over 10,000 simulations for true prevalence values 5%, 10% and 30% and true C-statistic of 0.64, 0.72, 0.8, 0.85 and 0.9

			C-statistic					Calibration slope					Calibration in the large				
p	C	n_e	SE_{true}	SE_{app}	%Bias SE_{app}	SE_{NI}	%Bias SE_{NI}	SE_{true}	SE_{app}	% Bias SE_{app}	SE_{NI}	% Bias SE_{NI}	SE_{true}	SE_{app}	% Bias SE_{app}	SE_{NI}	% Bias SE_{NI}
0.05	0.64	50	0.0405	0.0398	-2	0.0401	-1	0.295	0.290	-2	0.291	-1	0.145	0.146	1	0.146	1
		100	0.0283	0.0281	-1	0.0282	0	0.205	0.205	0	0.206	0	0.104	0.103	-1	0.103	-1
		200	0.0199	0.0199	0	0.0201	1	0.147	0.145	-1	0.145	-1	0.074	0.073	-2	0.073	-2
		400	0.0140	0.0141	0	0.0142	1	0.103	0.102	-1	0.103	-1	0.053	0.052	-2	0.052	-2
0.05	0.72	50	0.0372	0.0366	-2	0.0368	-1	0.188	0.183	-3	0.186	-1	0.148	0.148	0	0.148	0
		100	0.0260	0.0259	0	0.0259	0	0.134	0.129	-4	0.132	-2	0.106	0.104	-1	0.105	-1
		200	0.0184	0.0183	-1	0.0183	-1	0.093	0.091	-2	0.093	0	0.074	0.074	0	0.074	0
		400	0.0130	0.0129	-1	0.0129	0	0.066	0.064	-3	0.066	-1	0.053	0.052	-1	0.052	0
0.05	0.8	50	0.0318	0.0316	-1	0.0313	-2	0.142	0.131	-8	0.141	-1	0.155	0.154	-1	0.154	-1
		100	0.0225	0.0223	-1	0.0224	0	0.100	0.093	-7	0.100	0	0.109	0.109	0	0.109	0
		200	0.0157	0.0158	0	0.0158	0	0.069	0.065	-6	0.070	1	0.076	0.076	0	0.076	0
		400	0.0111	0.0112	0	0.0111	0	0.048	0.046	-5	0.050	2	0.054	0.054	-1	0.054	-1
0.05	0.85	50	0.0274	0.0275	0	0.0269	-2	0.126	0.110	-13	0.125	-1	0.160	0.161	1	0.159	0
		100	0.0192	0.0194	1	0.0191	-1	0.088	0.077	-12	0.088	1	0.113	0.114	1	0.113	0
		200	0.0135	0.0137	2	0.0135	0	0.062	0.055	-11	0.063	1	0.079	0.080	2	0.080	1
		400	0.0096	0.0097	1	0.0096	0	0.043	0.039	-10	0.044	3	0.056	0.057	1	0.056	0

0.05	0.9	50	0.0216	0.0225	4	0.0216	0	0.120	0.094	-21	0.118	-1	0.169	0.182	7	0.171
		100	0.0154	0.0159	3	0.0151	-2	0.084	0.067	-21	0.084	0	0.120	0.129	7	0.121
		200	0.0109	0.0112	3	0.0108	-1	0.058	0.047	-18	0.059	3	0.086	0.091	6	0.086
		400	0.0075	0.0080	6	0.0076	1	0.041	0.033	-19	0.042	2	0.060	0.064	7	0.061
0.1	0.64	50	0.0422	0.0409	-3	0.0410	-3	0.305	0.301	-1	0.302	-1	0.154	0.151	-2	0.151
		100	0.0290	0.0289	0	0.0290	0	0.215	0.213	-1	0.214	-1	0.107	0.106	-1	0.107
		200	0.0205	0.0204	0	0.0205	0	0.151	0.150	0	0.151	0	0.076	0.075	-1	0.075
		400	0.0145	0.0144	0	0.0145	0	0.106	0.106	0	0.107	0	0.053	0.053	0	0.053
0.1	0.72	50	0.0376	0.0376	0	0.0375	0	0.200	0.193	-4	0.197	-1	0.158	0.152	-4	0.154
		100	0.0264	0.0266	1	0.0265	0	0.142	0.135	-5	0.139	-3	0.108	0.107	-1	0.109
		200	0.0187	0.0188	0	0.0188	1	0.098	0.096	-2	0.099	1	0.077	0.076	-2	0.077
		400	0.0132	0.0133	0	0.0133	0	0.069	0.068	-1	0.070	1	0.054	0.054	-1	0.055
0.1	0.8	50	0.0324	0.0325	0	0.0324	0	0.157	0.142	-9	0.152	-3	0.163	0.154	-5	0.162
		100	0.0229	0.0229	0	0.0227	-1	0.108	0.100	-7	0.108	0	0.115	0.109	-6	0.114
		200	0.0163	0.0163	0	0.0162	-1	0.076	0.071	-7	0.076	0	0.080	0.077	-4	0.081
		400	0.0115	0.0115	0	0.0114	-1	0.053	0.050	-7	0.054	1	0.058	0.054	-6	0.057
0.1	0.85	50	0.0278	0.0282	1	0.0278	0	0.141	0.121	-14	0.137	-3	0.172	0.155	-10	0.169
		100	0.0196	0.0199	2	0.0195	-1	0.098	0.085	-13	0.097	-1	0.121	0.110	-9	0.119
		200	0.0138	0.0141	2	0.0138	0	0.067	0.060	-10	0.068	2	0.085	0.078	-8	0.084
		400	0.0097	0.0100	2	0.0098	1	0.048	0.043	-11	0.048	1	0.060	0.055	-9	0.060
0.1	0.9	50	0.0222	0.0229	3	0.0221	0	0.136	0.105	-22	0.130	-4	0.186	0.163	-12	0.181
		100	0.0156	0.0161	3	0.0157	1	0.091	0.074	-19	0.091	0	0.129	0.114	-11	0.127

		200	0.0110	0.0114	4	0.0109	-1	0.065	0.052	-19	0.065	0	0.091	0.081	-11	0.090
		400	0.0077	0.0081	5	0.0077	0	0.045	0.037	-18	0.046	1	0.065	0.057	-12	0.064
0.3	0.64	50	0.0481	0.0463	-4	0.0464	-4	0.369	0.351	-5	0.352	-5	0.179	0.174	-3	0.174
		100	0.0329	0.0327	-1	0.0328	0	0.251	0.248	-1	0.249	-1	0.125	0.123	-1	0.123
		200	0.0232	0.0231	0	0.0231	0	0.176	0.176	0	0.176	0	0.087	0.087	0	0.087
		400	0.0163	0.0164	0	0.0164	1	0.126	0.123	-2	0.124	-2	0.062	0.061	-1	0.061
0.3	0.72	50	0.0429	0.0426	-1	0.0425	-1	0.246	0.233	-5	0.235	-4	0.185	0.181	-2	0.181
		100	0.0302	0.0301	0	0.0302	0	0.172	0.164	-5	0.166	-4	0.128	0.128	0	0.128
		200	0.0214	0.0213	-1	0.0213	-1	0.121	0.116	-4	0.118	-3	0.090	0.090	0	0.091
		400	0.0150	0.0150	0	0.0151	1	0.084	0.082	-2	0.083	-1	0.064	0.064	0	0.064
0.3	0.8	50	0.0370	0.0367	-1	0.0364	-2	0.199	0.180	-10	0.186	-7	0.196	0.190	-3	0.194
		100	0.0260	0.0260	0	0.0258	-1	0.136	0.127	-6	0.132	-3	0.138	0.134	-3	0.137
		200	0.0184	0.0183	0	0.0183	-1	0.095	0.090	-5	0.093	-1	0.097	0.095	-3	0.097
		400	0.0130	0.0130	0	0.0129	-1	0.066	0.064	-4	0.066	0	0.069	0.067	-3	0.068
0.3	0.85	50	0.0317	0.0318	0	0.0316	0	0.185	0.160	-14	0.171	-8	0.208	0.191	-8	0.206
		100	0.0223	0.0225	0	0.0222	-1	0.123	0.113	-9	0.121	-2	0.146	0.135	-8	0.145
		200	0.0157	0.0159	1	0.0157	0	0.087	0.080	-8	0.085	-2	0.104	0.096	-8	0.103
		400	0.0111	0.0112	0	0.0112	0	0.060	0.056	-7	0.060	0	0.073	0.068	-7	0.073
0.3	0.9	50	0.0251	0.0253	1	0.0250	0	0.178	0.144	-19	0.163	-9	0.229	0.178	-22	0.223
		100	0.0177	0.0179	1	0.0177	0	0.121	0.102	-15	0.115	-5	0.162	0.126	-22	0.158
		200	0.0124	0.0127	2	0.0125	0	0.084	0.072	-14	0.081	-3	0.114	0.089	-22	0.112
		400	0.0088	0.0090	2	0.0088	0	0.058	0.051	-12	0.058	-1	0.081	0.063	-22	0.079

Table S6. DGM3. Number of events required to attain a specified standard error for $\hat{C}$, $\hat{\beta}_{cs}$ and $\hat{\alpha}_{CL}$. % Bias of the estimated sample size (and number of events) calculated over 10,000 simulations for true prevalence values 10% and 30% and true C-statistic of 0.64, 0.72, 0.8, 0.85 and 0.9.

		C-Statistic						Calibration Slope						Calibration in the large					
p	C	SE_{req}	$\hat{n}_{req}^{(e)}$	$\hat{n}_{req,ap}^{(e)}$	% Bias	$\hat{n}_{req,NI}^{(e)}$	% Bias	SE_{req}	$\hat{n}_{req}^{(e)}$	$\hat{n}_{req,app}^{(e)}$	% Bias	$\hat{n}_{req,NI}^{(e)}$	% Bias	SE_{req}	$\hat{n}_{req}^{(e)}$	$\hat{n}_{req,app}^{(e)}$	% Bias	$\hat{n}_{req,NI}^{(e)}$	% Bias
0.05	0.64	0.0125	513	507	-1	512	0	0.1	425	414	-3	422	-1	0.1	108	107	-1	107	-1
0.05	0.72	0.0125	415	427	3	424	2	0.1	176	165	-6	172	-2	0.1	111	110	-1	110	-1
0.05	0.80	0.0125	310	319	3	314	1	0.1	99	84	-15	94	-5	0.1	118	117	-1	117	-1
0.05	0.85	0.0125	235	237	1	231	-2	0.1	77	59	-23	74	-4	0.1	132	131	-1	125	-6
0.05	0.90	0.0125	139	153	11	144	4	0.1	66	42	-36	62	-6	0.1	165	164	-1	137	-17
0.10	0.64	0.0125	541	535	-1	534	-1	0.1	453	453	0	456	1	0.1	115	114	-1	114	-1
0.10	0.72	0.0125	447	451	1	449	0	0.1	198	186	-6	193	-2	0.1	120	116	-3	120	0
0.10	0.80	0.0125	329	339	3	333	1	0.1	118	100	-15	113	-4	0.1	130	118	-9	131	1
0.10	0.85	0.0125	241	251	4	242	0	0.1	94	72	-23	90	-4	0.1	146	121	-17	141	-3
0.10	0.90	0.0125	148	163	10	151	2	0.1	84	54	-36	79	-5	0.1	166	131	-21	157	-5
0.30	0.64	0.0125	695	687	-1	693	0	0.1	625	625	0	620	-1	0.1	153	152	-1	151	-1
0.30	0.72	0.0125	586	580	-1	578	-1	0.1	282	276	-2	277	-2	0.1	166	165	-1	166	0
0.30	0.80	0.0125	420	432	3	430	2	0.1	180	163	-9	173	-4	0.1	192	182	-5	191	0
0.30	0.85	0.0125	323	326	1	321	-1	0.1	149	128	-14	145	-2	0.1	216	183	-15	216	0
0.30	0.90	0.0125	205	209	2	206	0	0.1	147	105	-29	134	-9	0.1	240	160	-33	253	6

0.05	0.64	0.025	129	127	-2	129	0	0.15	192	187	-3	188	-2	0.15	49	48	-2	47	-3
0.05	0.72	0.025	108	107	-1	107	-1	0.15	77	75	-3	76	-1	0.15	50	49	-2	49	-2
0.05	0.80	0.025	78	80	3	79	1	0.15	46	39	-15	43	-7	0.15	53	52	-2	52	-2
0.05	0.85	0.025	58	60	3	56	-3	0.15	37	27	-27	33	-12	0.15	60	59	-2	56	-7
0.05	0.90	0.025	36	39	8	34	-6	0.15	32	19	-41	27	-14	0.15	74	73	-1	61	-17
0.10	0.64	0.025	136	134	-1	135	-1	0.15	209	199	-5	202	-3	0.15	52	51	-2	51	-2
0.10	0.72	0.025	112	113	1	113	1	0.15	89	83	-7	85	-4	0.15	55	52	-5	53	-3
0.10	0.80	0.025	86	85	-1	83	-3	0.15	54	45	-17	50	-7	0.15	58	53	-9	58	0
0.10	0.85	0.025	62	63	2	62	0	0.15	44	32	-27	40	-9	0.15	65	54	-17	63	-3
0.10	0.90	0.025	39	41	5	37	-5	0.15	40	25	-38	35	-12	0.15	73	58	-21	70	-4
0.30	0.64	0.025	173	172	-1	173	0	0.15	282	278	-1	275	-2	0.15	70	68	-3	67	-4
0.30	0.72	0.025	144	145	1	144	0	0.15	125	122	-2	123	-1	0.15	76	74	-3	74	-3
0.30	0.80	0.025	110	110	-2	107	-2	0.15	81	73	-10	77	-5	0.15	86	81	-6	85	-1
0.30	0.85	0.025	81	81	0	79	-2	0.15	70	57	-19	64	-8	0.15	97	82	-15	96	-1
0.30	0.90	0.025	52	53	2	50	-4	0.15	69	47	-32	60	-13	0.15	107	71	-34	112	5

Table S7. DGM3. Type I error and power to detect a statistically significant difference, d, from a target value of the C-statistic calculated over 10,000 simulations for true prevalence values 5%, 10% and 30% and true C-statistic of 0.64, 0.72, 0.8, 0.85 and 0.9 when $\hat{n}_{req,app}$ is chosen to give 90% power. $\hat{n}^{(e)}{}_{req,app}$ denotes the required number of events.

d	p	C_0	C_1	$\hat{n}^{(e)}{}_{\mathrm{req,app}}$	Type I error	Power
		0.64	0.67	762	0.054	0.903
0.03	0.05	0.72	0.75	626	0.043	0.902
		0.8	0.83	519	0.043	0.916
		0.64	0.67	777	0.052	0.896
0.03	0.10	0.72	0.75	667	0.045	0.905
		0.8	0.83	552	0.045	0.913
		0.64	0.67	1015	0.05	0.903
0.03	0.30	0.72	0.75	870	0.042	0.914
		0.8	0.83	674	0.043	0.917
		0.64	0.69	263	0.049	0.901
0.05	0.05	0.72	0.77	229	0.045	0.911
		0.8	0.85	175	0.04	0.912
		0.64	0.69	286	0.047	0.909
0.05	0.10	0.72	0.77	245	0.047	0.914
		0.8	0.85	192	0.045	0.92
		0.64	0.69	355	0.049	0.902
0.05	0.30	0.72	0.77	311	0.047	0.911
		0.8	0.85	236	0.045	0.921

Table S8. DGM4. % Bias of $SE_{app}(\hat{C})$, $SE_{app}(\hat{\beta}_{CS})$, $SE_{app}(\hat{\alpha}_{CL})$, $SE_{MC}(\hat{\beta}_{CS})$ and $SE_{MC}(\hat{\alpha}_{CL})$ calculated over 10,000 simulations for true prevalence values 5%, 10% and 30% and true C-statistic of 0.64, 0.72, 0.8, 0.85 and 0.9.

			C-Statistic			Calibration Slope					Calibration in the large				
p	C	n_{events}	SE_{true}	SE_{app}	% Bias SE_{app}	SE_{true}	SE_{app}	% Bias SE_{app}	SE_{NI}	% Bias SE_{NI}	SE_{true}	SE_{app}	% Bias SE_{app}	SE_{NI}	% Bias SE_{NI}
0.05	0.64	50	0.043	0.040	-5	0.267	0.293	10	0.293	10	0.151	0.148	-2	0.148	-2
		100	0.030	0.029	-4	0.185	0.208	12	0.210	13	0.106	0.105	-2	0.105	-1
		200	0.021	0.020	-4	0.131	0.147	12	0.148	12	0.076	0.074	-2	0.074	-2
		400	0.015	0.014	-4	0.092	0.104	13	0.104	13	0.053	0.052	-2	0.052	-1
0.05	0.72	50	0.038	0.036	-5	0.171	0.180	5	0.184	8	0.148	0.146	-1	0.146	-1
		100	0.027	0.025	-4	0.119	0.127	7	0.130	9	0.104	0.103	-1	0.103	0
		200	0.019	0.018	-4	0.084	0.090	7	0.092	9	0.072	0.073	-1	0.073	1
		400	0.013	0.013	-5	0.060	0.064	7	0.065	9	0.052	0.051	1	0.052	0
0.05	0.8	50	0.034	0.031	-8	0.125	0.127	2	0.138	10	0.158	0.153	-1	0.153	-3
		100	0.024	0.022	-8	0.088	0.090	3	0.096	10	0.110	0.108	-3	0.108	-2
		200	0.017	0.016	-8	0.062	0.064	3	0.069	12	0.078	0.076	-2	0.076	-2
		400	0.012	0.011	-7	0.043	0.045	4	0.049	13	0.055	0.054	-2	0.054	-1
0.05	0.85	50	0.031	0.028	-12	0.111	0.110	0	0.125	13	0.165	0.161	-2	0.159	-3
		100	0.022	0.020	-12	0.078	0.078	1	0.089	14	0.116	0.114	-2	0.112	-3
		200	0.016	0.014	-11	0.055	0.055	0	0.063	14	0.083	0.080	-2	0.080	-4
		400	0.011	0.010	-11	0.038	0.039	2	0.045	17	0.058	0.057	-3	0.057	-2
0.05	0.9	50	0.025	0.021	-17	0.098	0.089	-10	0.113	15	0.174	0.171	-2	0.164	-6

		100	0.017	0.015	-16	0.068	0.063	-7	0.080	18	0.121	0.121	-2	0.116	-5
		200	0.012	0.010	-17	0.048	0.044	-8	0.057	18	0.086	0.086	0	0.082	-5
		400	0.009	0.007	-17	0.034	0.031	-7	0.040	19	0.061	0.061	-1	0.058	-5
0.1	0.64	50	0.041	0.040	-3	0.268	0.287	7	0.289	8	0.151	0.148	-1	0.149	-2
		100	0.030	0.028	-4	0.188	0.204	9	0.205	9	0.106	0.105	-2	0.105	-1
		200	0.021	0.020	-3	0.131	0.144	10	0.145	10	0.075	0.074	-1	0.074	-1
		400	0.015	0.014	-4	0.094	0.102	8	0.102	9	0.052	0.052	-1	0.053	0
0.1	0.72	50	0.039	0.038	-4	0.184	0.192	4	0.195	6	0.158	0.152	0	0.155	-2
		100	0.028	0.027	-5	0.129	0.135	4	0.138	7	0.111	0.108	-3	0.109	-2
		200	0.020	0.019	-5	0.090	0.095	6	0.097	8	0.079	0.076	-3	0.078	-2
		400	0.014	0.013	-4	0.064	0.068	6	0.069	8	0.055	0.054	-3	0.055	0
0.1	0.8	50	0.035	0.032	-7	0.141	0.140	0	0.151	7	0.164	0.153	-2	0.161	-2
		100	0.024	0.023	-6	0.098	0.099	2	0.107	10	0.117	0.108	-7	0.114	-3
		200	0.017	0.016	-7	0.069	0.070	2	0.076	10	0.084	0.076	-8	0.081	-4
		400	0.012	0.011	-6	0.048	0.050	3	0.053	11	0.059	0.054	-9	0.057	-3
0.1	0.85	50	0.030	0.027	-10	0.124	0.118	-5	0.135	8	0.178	0.152	-8	0.167	-6
		100	0.021	0.019	-10	0.086	0.084	-2	0.095	11	0.122	0.108	-12	0.118	-3
		200	0.015	0.014	-9	0.061	0.059	-3	0.067	11	0.087	0.076	-12	0.083	-4
		400	0.011	0.010	-10	0.043	0.042	-2	0.048	12	0.062	0.054	-13	0.059	-4
0.1	0.9	50	0.026	0.022	-15	0.115	0.102	-11	0.127	10	0.200	0.160	-20	0.180	-10
		100	0.018	0.015	-15	0.080	0.072	-9	0.090	13	0.139	0.113	-19	0.127	-9
		200	0.013	0.011	-16	0.055	0.051	-7	0.064	15	0.098	0.080	-18	0.090	-9

		400	0.009	0.008	-16	0.039	0.036	-7	0.045	16	0.070	0.057	-19	0.064	-9
0.3	0.64	50	0.047	0.046	-3	0.346	0.351	1	0.351	1	0.173	0.172	-1	0.172	-1
		100	0.033	0.033	-1	0.239	0.249	4	0.250	4	0.125	0.122	-2	0.122	-2
		200	0.023	0.023	-1	0.167	0.176	5	0.177	6	0.087	0.086	0	0.086	0
		400	0.016	0.016	0	0.117	0.125	7	0.125	7	0.062	0.061	-2	0.061	-2
0.3	0.72	50	0.043	0.042	-3	0.232	0.230	-1	0.233	1	0.183	0.180	-2	0.180	-2
		100	0.030	0.030	-2	0.159	0.163	3	0.165	4	0.130	0.127	-2	0.127	-1
		200	0.021	0.021	-2	0.111	0.115	4	0.117	5	0.091	0.090	-1	0.090	-2
		400	0.015	0.015	-2	0.079	0.081	3	0.082	4	0.064	0.064	-1	0.064	-1
0.3	0.8	50	0.039	0.037	-4	0.191	0.182	-4	0.189	-1	0.198	0.189	-4	0.193	-1
		100	0.027	0.026	-3	0.131	0.128	-2	0.133	2	0.140	0.134	-4	0.137	-2
		200	0.019	0.019	-3	0.091	0.091	0	0.094	4	0.099	0.095	-5	0.097	-3
		400	0.013	0.013	-2	0.063	0.064	1	0.067	5	0.070	0.067	-4	0.068	-3
0.3	0.85	50	0.033	0.032	-4	0.181	0.160	-11	0.171	-5	0.215	0.192	-11	0.205	-2
		100	0.023	0.023	-4	0.122	0.113	-8	0.121	-1	0.151	0.136	-10	0.145	-5
		200	0.016	0.016	-3	0.084	0.080	-5	0.085	1	0.105	0.096	-9	0.103	-4
		400	0.012	0.011	-3	0.059	0.056	-4	0.060	3	0.075	0.068	-10	0.073	-3
0.3	0.9	50	0.028	0.026	-8	0.174	0.145	-17	0.164	-6	0.236	0.177	-25	0.224	-4
		100	0.020	0.018	-9	0.116	0.102	-11	0.116	0	0.167	0.125	-25	0.158	-5
		200	0.014	0.013	-7	0.080	0.072	-10	0.082	2	0.119	0.088	-26	0.112	-5
		400	0.010	0.009	-8	0.056	0.051	-8	0.058	4	0.085	0.062	-27	0.079	-6

Table S9. Survival data –Random Censoring. % Bias of $SE_{app}(\hat{C})$ and $SE_{app}(\hat{\beta}_{CS})$ obtained from the formulae for binary data and calculated over 10,000 simulations for true prevalence values 5%, 10% and 30% and true C-index of 0.64, 0.72, 0.8, 0.85 and 0.9.

			C-statistic			Calibration Slope		
p	C	n_{events}	SE_{true}	SE_{app}	% Bias (SE_{app})	SE_{true}	SE_{app}	% Bias (SE_{app})
0.05	0.64	50	0.0443	0.0396	-11	0.2750	0.2820	3
		100	0.0322	0.0280	-13	0.2000	0.1990	-1
		200	0.0219	0.0198	-10	0.1360	0.1410	4
		400	0.0158	0.0140	-11	0.0970	0.1000	3
0.05	0.72	50	0.0410	0.0364	-11	0.1790	0.1800	1
		100	0.0288	0.0256	-11	0.1230	0.1270	3
		200	0.0202	0.0181	-10	0.0860	0.0900	5
		400	0.0142	0.0128	-10	0.0610	0.0630	3
0.05	0.80	50	0.0336	0.0308	-8	0.1270	0.1270	0
		100	0.0240	0.0218	-9	0.0870	0.0900	3
		200	0.0167	0.0154	-8	0.0610	0.0630	3
		400	0.0119	0.0109	-8	0.0430	0.0450	5
0.05	0.85	50	0.0287	0.0264	-8	0.1090	0.1075	-1
		100	0.0199	0.0187	-6	0.0742	0.0754	2
		200	0.0141	0.0132	-6	0.0522	0.0532	2
		400	0.01	0.0094	-6	0.0364	0.0375	3
0.05	0.90	50	0.0223	0.0219	-2	0.0976	0.0920	-6
		100	0.0155	0.0154	-1	0.0661	0.0649	-2
		200	0.0111	0.0109	-2	0.0464	0.0458	-1
		400	0.0078	0.0077	-2	0.0326	0.0324	-1
0.10	0.64	50	0.0457	0.0412	-10	0.3020	0.3110	3
		100	0.0317	0.0291	-8	0.2060	0.2180	6
		200	0.0222	0.0205	-8	0.1430	0.1540	8
		400	0.0157	0.0145	-8	0.1010	0.1090	8
0.10	0.72	50	0.0403	0.0367	-9	0.1790	0.1880	5
		100	0.0278	0.0259	-7	0.1230	0.1320	7
		200	0.0197	0.0183	-7	0.0870	0.0930	7
		400	0.0138	0.0129	-7	0.0610	0.0660	8
0.10	0.80	50	0.035	0.0331	-5	0.1380	0.1460	6
		100	0.0247	0.0234	-5	0.0950	0.1030	8
		200	0.0175	0.0165	-6	0.0660	0.0730	11
		400	0.0123	0.0117	-5	0.0470	0.0510	9
0.10	0.85	50	0.0282	0.0276	-2	0.1131	0.1204	6
		100	0.0199	0.0195	-2	0.0783	0.0846	8
		200	0.0139	0.0138	-1	0.0549	0.0596	9
		400	0.0099	0.0098	-1	0.0382	0.0421	10

		50	0.0215	0.0223	4	0.1007	0.1040	3
0.10	0.90	100	0.0150	0.0157	5	0.0684	0.0731	7
		200	0.0107	0.0111	4	0.0474	0.0517	9
		400	0.0074	0.0079	7	0.0336	0.0365	9
		50	0.0453	0.0431	-5	0.2960	0.3250	10
0.20	0.64	100	0.0313	0.0305	-3	0.2020	0.2290	13
		200	0.0223	0.0216	-3	0.1420	0.1610	13
		400	0.0158	0.0153	-3	0.0990	0.1130	14
		50	0.0397	0.0393	-1	0.1850	0.2130	15
0.20	0.72	100	0.0276	0.0278	1	0.1280	0.1490	16
		200	0.0194	0.0196	1	0.0880	0.1050	19
		400	0.0138	0.0139	1	0.0620	0.0740	19
		50	0.0325	0.0336	3	0.1340	0.1580	18
0.20	0.80	100	0.0229	0.0237	3	0.0930	0.1110	19
		200	0.0163	0.0168	3	0.0650	0.0780	20
		400	0.0112	0.0118	5	0.0450	0.0550	22
		50	0.0276	0.0294	7	0.1205	0.1420	18
0.20	0.85	100	0.0193	0.0208	8	0.0826	0.0996	21
		200	0.0136	0.0147	8	0.0569	0.0701	23
		400	0.0096	0.0104	8	0.0400	0.0495	24
		50	0.0205	0.0234	14	0.1099	0.1269	15
0.20	0.90	100	0.0143	0.0167	17	0.0736	0.0885	20
		200	0.0100	0.0118	18	0.0511	0.0625	22
		400	0.0071	0.0083	17	0.0358	0.0442	23

Figure S1. Standard errors for the estimated calibration slope from LDA and logistic regression

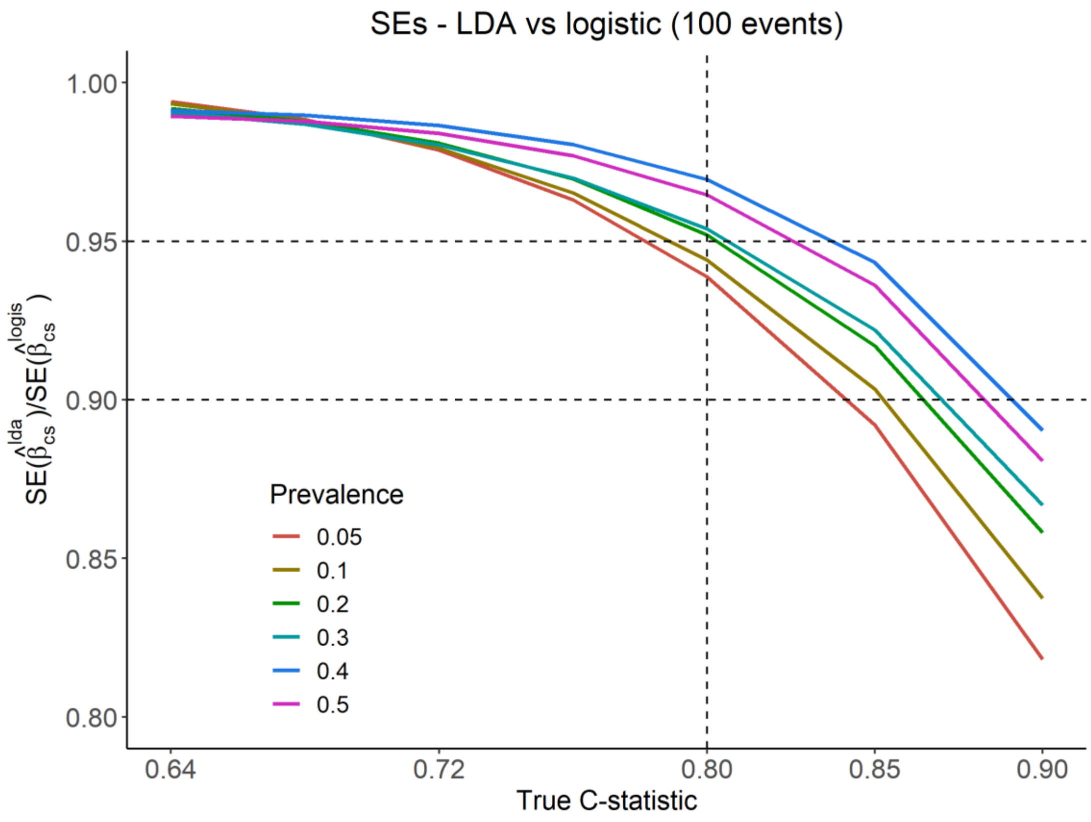

Figure S2. Marginal distribution of the linear predictor given the outcome under DGM1 when prevalence equals 0.1.

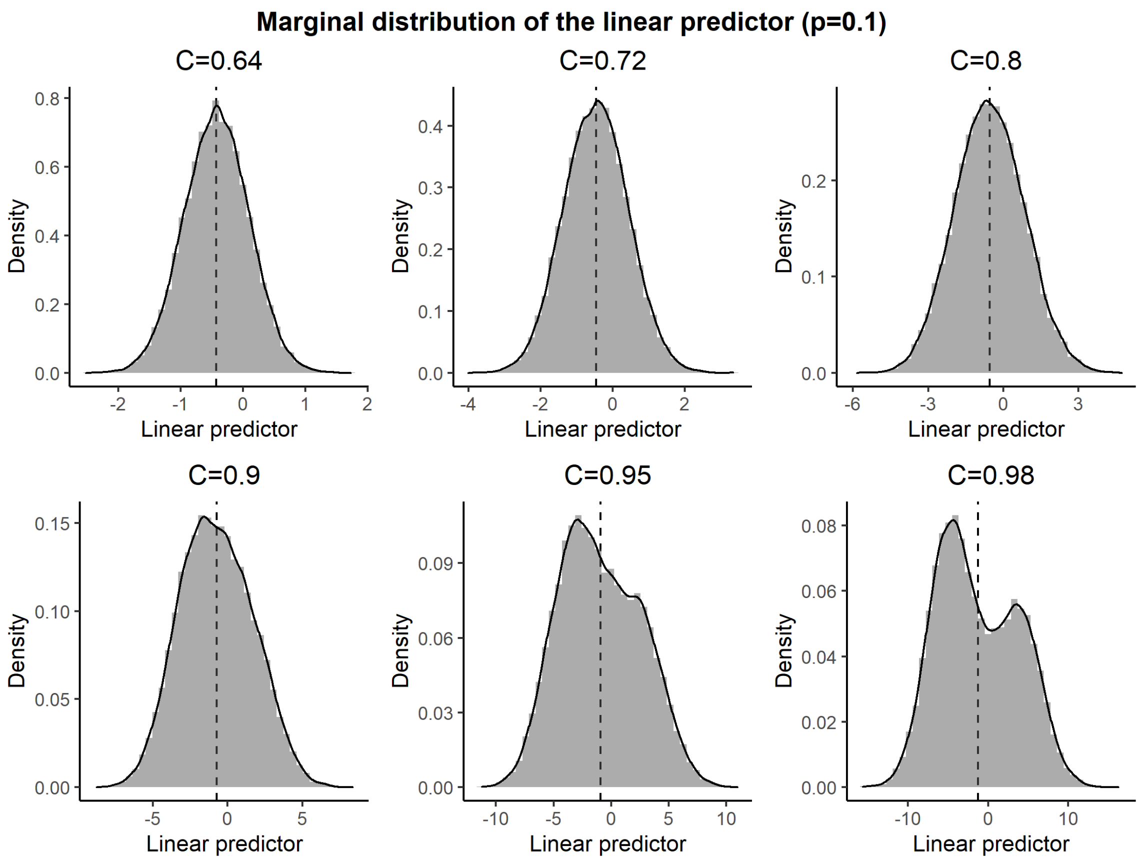

Figure S3. Conditional distribution of the linear predictor given the outcome under DGM3 when prevalence equals 0.1.

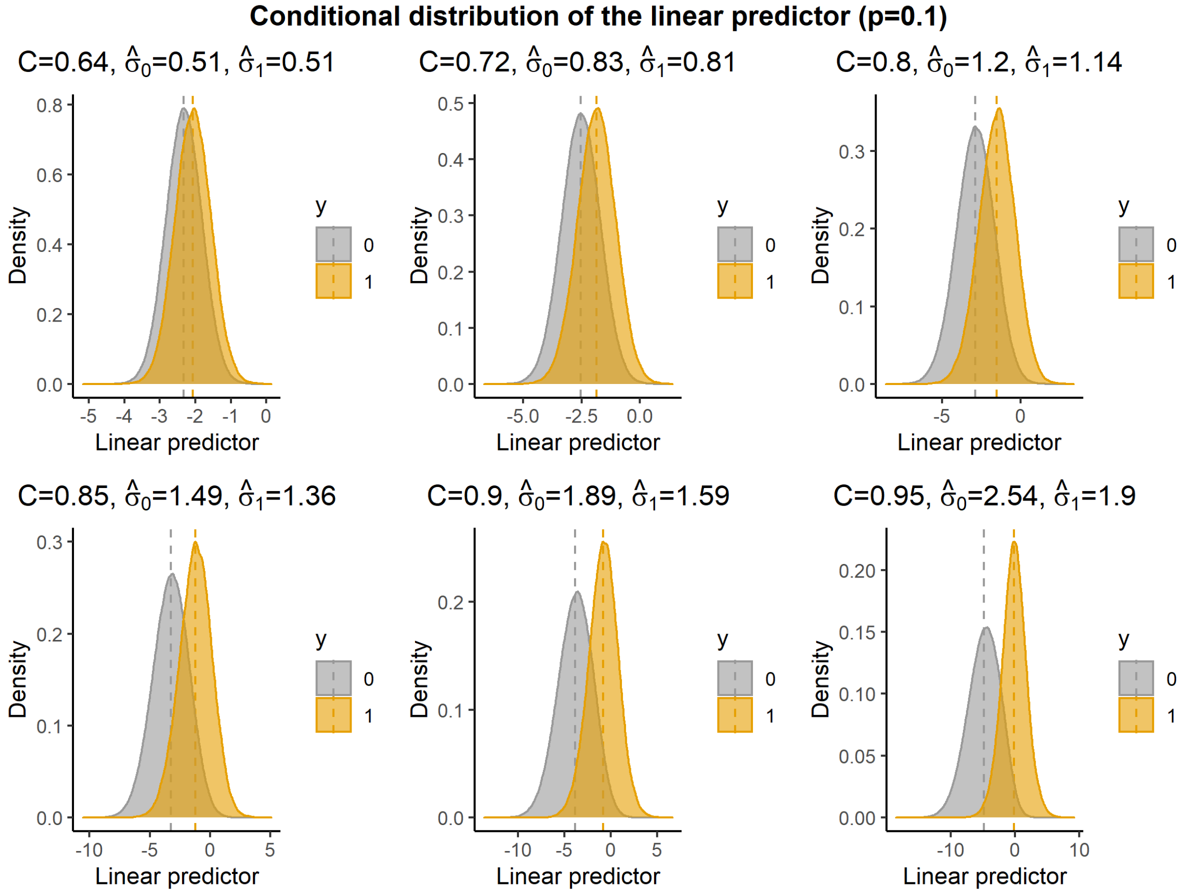

Figure S4. Conditional distribution of the linear predictor given the outcome under DGM4 when prevalence equals 0.1

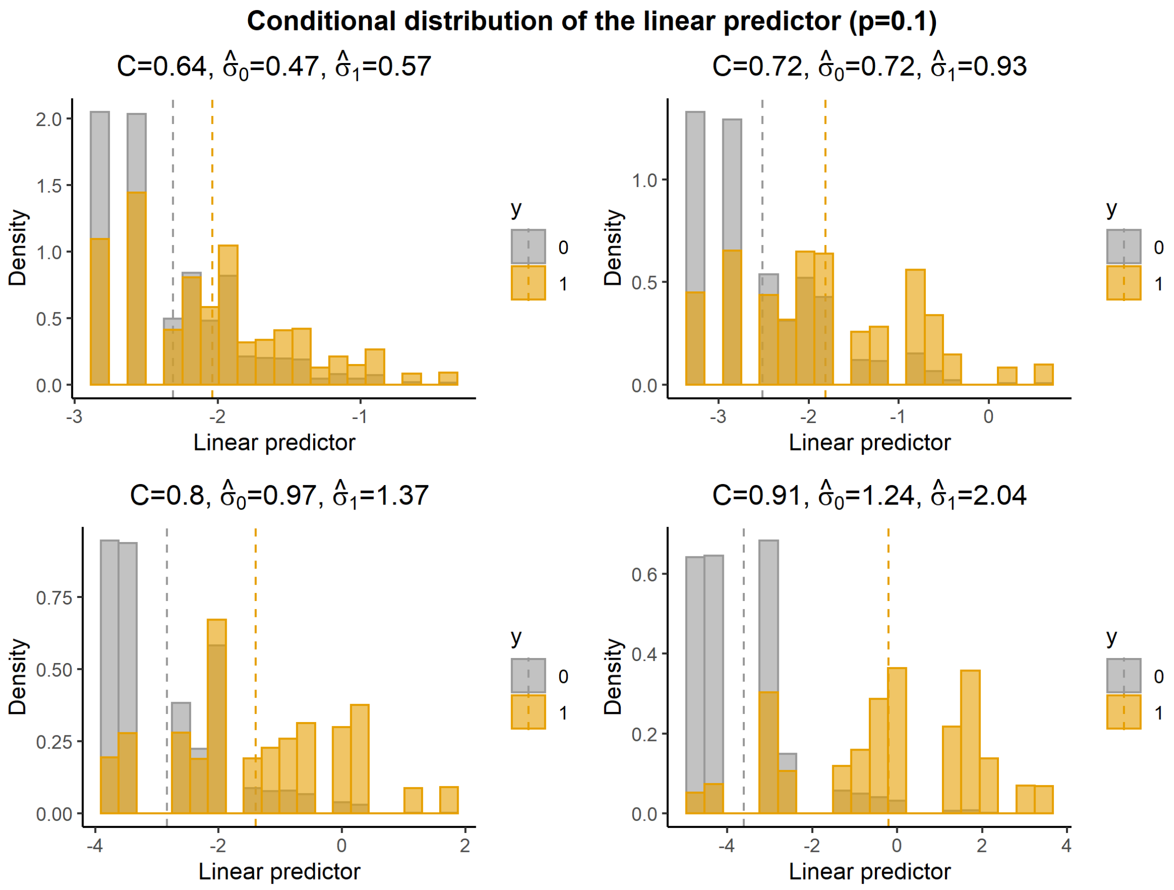

Supplement: sj-pdf-2-smm-10.1177_09622802211007522 - Supplemental material for Estimation of required sample size for external validation of risk models for binary outcomes [file sj-pdf-2-smm-10.1177_09622802211007522.pdf]
